# *In Silico* Exploration for Novel Type-I Inhibitors of Tie-2/TEK: The Performance of Different Selection Strategy in Selecting Virtual Screening Candidates

**DOI:** 10.1038/srep37628

**Published:** 2016-11-23

**Authors:** Peichen Pan, Huiyong Sun, Hui Liu, Dan Li, Wenfang Zhou, Xiaotian Kong, Youyong Li, Huidong Yu, Tingjun Hou

**Affiliations:** 1 College of Pharmaceutical Sciences, Zhejiang University, Hangzhou, Zhejiang 310058, China; 2 Institute of Functional Nano and Soft Materials (FUNSOM), Soochow University, Suzhou, Jiangsu 215123, China; 3 Rongene Pharma Co., Ltd., International Business Incubator, Guangzhou Science Town, Guangdong 510663, China; 4 State Key Lab of CAD&CG, Zhejiang University, Hangzhou, Zhejiang 310058, China

## Abstract

The receptor tyrosine kinase Tie-2 is involved in vessel remodeling and maturation, and has been regarded as a potential target for the treatment of various solid tumors. The absence of novel, potent and selective inhibitors severely hampers the understanding of the therapeutic potential of Tie-2. In the present work, we describe the discovery of novel type-I inhibitors of Tie-2 by structure-based virtual screening. Preliminary SAR was also performed based on one active compound, and several novel inhibitors with low micro-molar affinity were discovered. To directly compare the efficiency between different filtering strategies in selecting VS candidates, two methods were separately carried out to screen the same chemical library, and the selected VS candidates were then experimentally assessed by *in vitro* enzymatic assays. The results demonstrate that the hit rate is improved when stricter drug-likeness criteria and less number of molecules for clustering analysis are used, and meanwhile, the molecular diversity of the compounds still maintains. As a case study of TIE-2, the information presented in this work underscores the importance of selecting an appropriate selection strategy in VS campaign, and the novel inhibitors identified and the detailed binding modes of action provide a starting point for further hit-to-lead optimization process.

Angiogenesis is involved in the formation of new capillaries from existing vasculature, where a primitive vascular network is assembled due to the differentiation and proliferation of endothelial cells^1^. The activation of angiogenesis usually occurs in embryonic development but can also be found in normal physiological processes such as wound healing and certain stages of the menstrual cycle. Aberrant angiogenesis is demonstrated to be the cause of numerous life-threatening diseases including cancer, inflammatory disorders, ischemic diseases and various retinopathies^2^. The growth of tumors has been shown to rely on the progress of angiogenesis, and interference with the formation of vascular system is believed to be an effective strategy for the treatment of various solid tumors^3^.

Great success has been made in the development of drugs targeting angiogenesis signaling pathways in the past years. The members of the vascular endothelial cell growth factor (VEGF) and tyrosine kinase with immunoglobulin and epidermal growth factor homology domains-2 (Tie-2) have been shown to be essential factors in vascular development. The VEGF family, such as Flt-1 (VEGF-R1), Flk-1/KDR (VEGF-R2), and Flt-4 (VEGF-R3), plays critical roles in the sprouting process of angiogenesis^4^,^5^. While, Tie-2 receptors have been implicated in further stabilization, maturation and remodeling of preliminary vessels^6^,^7^,^8^. It is currently well established that blockage of VEGF and/or Tie-2 signaling pathways can significantly attenuate tumor-induced angiogenesis and suppress tumor growth and metastasis in a variety of solid tumors. Numerous anti-angiogenesis inhibitors targeting VEGF signaling have been under clinical assessment, and several of them, such as sunitinib (Sutent) and sorafenib (Nexavar), have been approved by the Food and Drug Administration (FDA)^4^,^9^,^10^. Despite of the encouraging clinical outcomes from VEGF inhibitors, more patients emerge to become resistant to currently available drugs^11^,^12^,^13^,^14^,^15^.

The emergence of drug resistance and the increasing need for better therapeutic strategies lead to the development of second-generation anti-angiogenesis drugs targeting different stages of vessel growth. Among them, angiopoietins (ANGs) and their physiologic receptors, such as Tie-2/TEK receptor that is expressed principally on vascular endothelium, have become very promising therapeutic targets. A substantial body of evidence has been reported that combination of different anti-angiogenesis inhibitors can obtain superior therapeutic outcomes compared with treatment using either agent alone in a variety of xenograft models^16^,^17^. Several inhibitory antibodies of ANGs (ANG-1 and/or ANG-2), such as Trebananib, MEDI-3617, CVX-060, REGN-910 and AMG-780^18^,^19^,^20^, have entered clinical trials, but the development of selective small-molecule inhibitors of Tie-2 is still in urgent need with only two candidates with poor kinase selectivity in early clinical phase, i.e. CEP-11981 (Phase I, Cephalon, Inc.) and ARRY-614 (Phase I, Array Biopharma. Inc.)^21^,^22^. However, hundreds of molecules were found effect in inhibiting Tie-2 activity including some FDA-approved tyrosine kinase inhibitors, several of which showed quite good inhibitory activity in nM level. Though these inhibitors may not be developed specially towards Tie-2, they are still helpful in understanding the binding patterns of Tie-2 and offer clues for the design of selective Tie-2 inhibitors.

Structure-based virtual screening (VS) strategy has been successfully applied in identifying novel inhibitors of a specific protein target^23^,^24^,^25^,^26^,^27^,^28^,^29^,^30^. However, the prediction accuracy of molecular docking and the percentage rate of active compounds are always low. Except for the influence of docking simulations, the effect of applying different selection strategy in selecting VS candidates is also obvious. Though numerous attempts have been made to improve the efficiency of VS in *in silico* models, few approaches were experimentally validated^27^,^31^,^32^. In the present work, structure-based VS was performed to identify type-I inhibitors of Tie-2 using different drug-likeness filtering criteria (Fig. 1). In VS campaign, clustering analysis can be performed based on the top ranked compounds to maximize the molecular diversity of the candidates. But this practice may have a pronounced influence on the overall hit rates. So we also assessed the influence of different number of clustering compounds on the overall hit rates by using the same docking method (Glide docking) and chemical library (ChemBridge), and two groups of VS candidates obtained from different clustering strategies were then experimentally evaluated. Afterwards, substructure searching was carried out to optimize the active compound (TP-C2-9, IC_50_ = 1.86 μM) by introducing different substitutions on the core structure and to give a preliminary structure-activity relationships (SAR) analysis. The activities of the compounds discovered in this work are all in low μM range, which is much higher than some already known inhibitors, but these novel structures could be a starting point for medicinal chemists to optimize the activity and benefit to developing more potent and selective Tie-2 inhibitors. Molecular modeling of the active compounds proposed the binding mode to Tie-2. Our results demonstrate that the hit rates can be well improved when stricter drug-likeness criteria are used and top-200 rather than top-1000 compounds are included in clustering analysis, and meanwhile, the molecular diversity of the compounds still maintains. The information provided and the active compounds identified in this work can help to guide the design and discovery of novel and potent inhibitors of Tie-2 as well as the structure-based VS of other protein targets.

## Results and Discussions

### Structure-based virtual screening

At present, two DFG-in structures (Type-I) of Tie-2 complexes (PDB entries: 2WQB and 3L8P)^33^,^34^ are available in RCSB Protein Data Bank^35^. Both crystal structures were firstly assessed for their ability to distinguish the known inhibitors from non-inhibitors of Tie-2 using two different scoring modes of docking (Glide SP and XP). The distributions of the docking scores for both inhibitors and non-inhibitors were plotted in Fig. 2. The student’s t-test was used to assess the significance of the difference between two sets of docking scores with the associated probability (*p* value). Moreover, the AUC values (area under curve) of the ROC (receiver operating characteristic) curves for both crystals based on the docking scores were also plotted in Supplementary Fig. S3 to quantitatively evaluate the prediction accuracy. We can find that the *p* values for 3L8P are much lower than those for 2WQB and the AUC values from SP and XP docking of 3L8P crystal are both higher than those from 2WQB, indicating that the discrimination power of molecular docking based on 3L8P is much higher. Thus, the crystal structure of 3L8P was employed in our VS campaign. For both 2WQB and 3L8P crystals, the performance of Glide XP docking is slightly better than that of SP docking, but the computational cost of XP docking is relatively much higher. Therefore, the Glide SP docking was utilized in the first-round screening of the entire ChemBridge database (~1,020,000 compounds). Afterwards the top-ranked 100,000 compounds from the SP docking were submitted to the second-round screening using the Glide XP docking, and the top-ranked 2,000 compounds were then saved.

**Figure 1:**
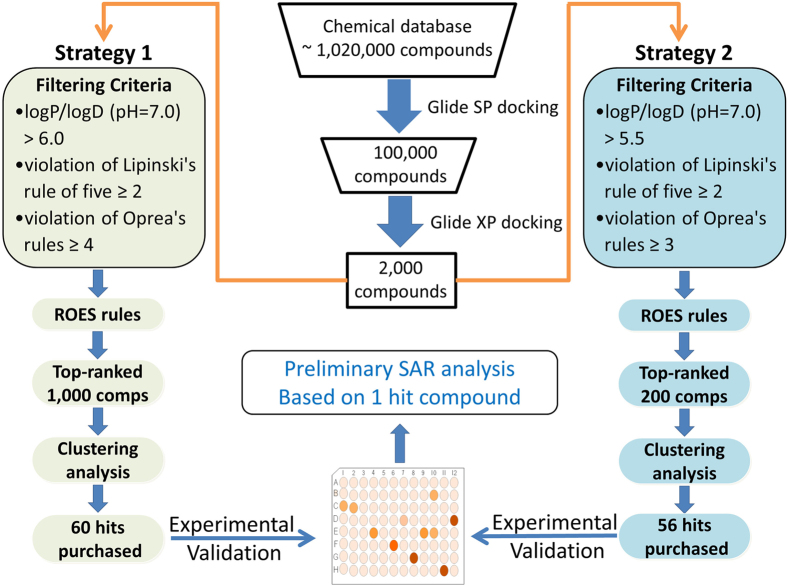
Graphical representation of the VS workflow.

**Figure 2:**
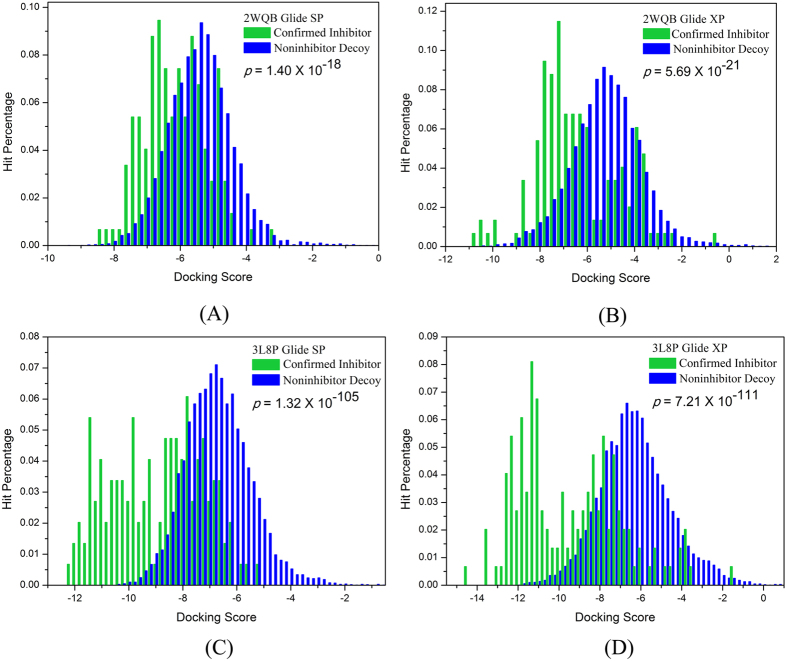
Distributions of the docking scores for the inhibitors and non-inhibitors based on (**A**) 2WQB, Glide SP docking, (**B**) 2WQB, Glide XP docking, (**C**) 3L8P, Glide SP docking and (**D**) 3L8P, Glide XP docking.

In order to assess the influence of different criteria in selecting VS candidates on the final hit percentage of the active compounds, two selection strategies were separately carried out to filter the same top-ranked 2,000 compounds from the Glide XP docking (Fig. 1). The ADMET properties of the 2,000 compounds were firstly predicted by ACD/ADME package^36^. As we can see in Fig. 1, two different drug-likeness criteria were used: (1) log*P*/log*D* (pH = 7.0) > 6.0; violation number of Lipinski’s rule of five ≥ 2^37^; violation number of Oprea’s rules of drug-likeness ≥ 4^38^. (2) log*P*/log*D* (pH = 7.0) > 5.5; violation number of Lipinski’s rule of five ≥ 2^37^; violation number of Oprea’s rules of drug-likeness ≥ 3^38^. All the molecules satisfying either criterion for each group were removed. The remaining compounds from both groups were then filtered by ROES rules to remove molecules containing toxic, reactive, and undesirable functional groups. For **Strategy 1**, the top-ranked 1,000 compounds were submitted to clustering analysis to maximize the structural diversity on the basis of the Tanimoto distance predicted from the FCFP_4 fingerprints. For **Strategy 2**, clustering analysis was performed with only the top-ranked 200 compounds. The Tanimoto Coefficient (tanimoto) metric was used for clustering based on the FCFP_4 fingerprints of the compounds in DS 2.5. The same number of 70 clusters was manually defined for each strategy prior to the clustering analysis, and the compounds were extracted using the method of selecting the cluster centers (a maximum dissimilarity method) to find diverse molecules^39^. Finally, almost the same number of molecules was obtained from each strategy. However some compounds were not available for purchasing from the ChemBridge vendor. So eventually 60 compounds were purchased from **Strategy 1** and 56 compounds were purchased from **Strategy 2**. The inhibitory activities of these compounds were then validated by biological experimental assays. Based on the most potent compound, structural optimization was also carried out to give a preliminary structure activity relationship (SAR) analysis using substructure searching method by querying the entire ChemBridge library for compounds with the same core structure.

### The performance of different filtering strategies in selecting VS candidates

**Figure 3:**
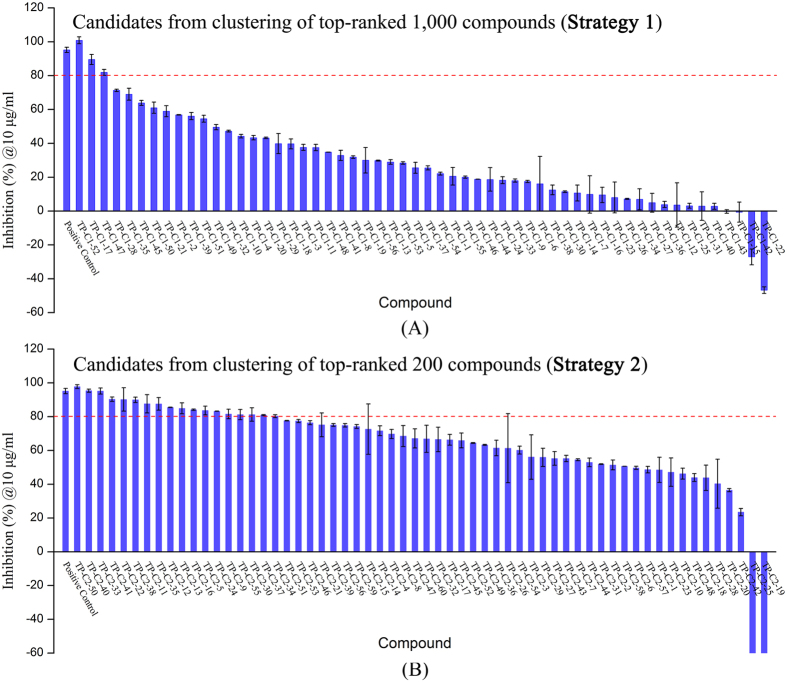
Inhibition rates (%) of the candidates from two filtering strategies at the concentration of 10 μg/mL. The compounds were tested in duplicate or more for every compound concentration.

**Figure 4:**
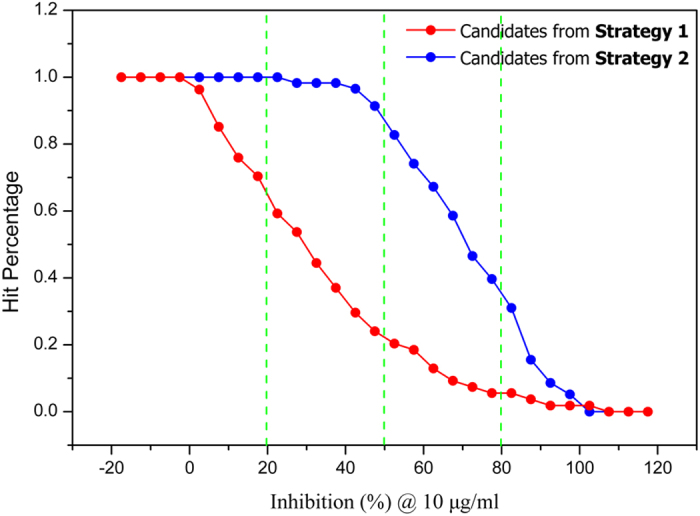
Comparison of hit percentage between candidates from two filtering strategies with different cutoff values of inhibition rates.

In order to assess the biological inhibitory activity of the VS compounds, Z’-Lyte enzymatic activity assay based on FRET (Fluorescence Resonance Energy Transfer) technology was developed as described above. The Tie-2 inhibitor (CAS: 948557–43–5) was previously reported as a tool inhibitor that can potently inhibit Tie-2 activity with an IC_50_ value of 0.25 μM, and was used as a positive control inhibitor in our study. The tested inhibitory activity of this Tie-2 inhibitor is 0.31 μM, which is in good agreement with the ref. 40. At first, all the compounds obtained from both filtering strategies were preliminary screened for their ability to inhibit Tie-2 kinase activity at the concentration of 10 μg/mL. The results were summarized and plotted in Fig. 3 and Fig. 4. It is easy to find that the performance of the candidates chosen from the clustering of the top-ranked 200 compounds is better than those chosen from the clustering of the top-ranked 1,000 compounds. The probability p value of the two sets is 0.00018 indicating that they are statistically different. As we can see in Fig. 4, over 80 percentage of the purchased compounds from **Strategy 2** were found to inhibit ≥50% Tie-2 activity at 10 μg/mL, and ~40% compounds can inhibit ≥80% Tie-2 activity. However, only ~20% compounds from **Strategy 1** were observed to inhibit ≥50% Tie-2 activity, and <10% compounds were found to inhibit ≥80% activity. This indicates that the hit rate from **Strategy 2** is much higher than that from **Strategy 1**. Different selection strategies in selecting the same 2,000 VS candidates have a pronounced influence on the overall hit rates of the active compounds. The results demonstrate that the hit rates can be well improved when stricter drug-likeness criteria and top-200 rather than top-1000 compounds for clustering analysis are used.

In fact, different drug-likeness criteria may affect the ADMET properties of the hit compounds, but may have little influence on the inhibitory activity. So, further analyses were carried out based on the VS result. We find that only six compounds (TP-C1-4, TP-C1-6, TP-C1-13, TP-C1-18, TP-C1-20, TP-C1-31) from Strategy 1 were filtered out using the criteria applied in **Strategy 2**. Among them, TP-C1-4 is active and the others are inactive. So the final hit rate of **Strategy 1** is only slightly influenced when the criteria of **Strategy 2** are applied. The likelihood of the difference based on the new data sets with the six compounds excluded from the list was also calculated, and the p value is 0.0002 implying that there is also a statistical difference between the two sets. Moreover, a power analysis to see how many compounds are needed to test to be confident was also performed and the results were summarized in Supplementary Table S2. A sample size of 50 is sufficient to get a power of 0.939, which says we have a 93% chance of detecting a difference if it collects 50 compounds. From this point of view, the high hit rates from Strategy 2 should be mainly contributed by using different clustering strategies. However stricter drug-likeness filtering criteria are also suggested since the potential druggability of the discovered hit compounds is somewhat better. In addition, the rankings of the tested compounds from both **Strategy 1** and **Strategy 2** were also summarized and plotted (see Supplementary Fig. S2). We can see that the rankings of all the compounds obtained from **Strategy 2** are within 200, and only a limited number of compounds from **Strategy 1** are within top-ranked 200, which is in part responsible for the observed difference in activity. Furthermore, the rankings of the compounds with >60% inhibitory activity @ 10 μg/ml from **Strategy 1** are 132, 147, 189, 375, 38 and 252 respectively, which are all higher than 400. In other words, no compounds with rankings lower than 400 are found potent inhibitors. The rankings of two most potent inhibitors, TP-C1-17 and TP-C1-47 showing <10 μM activity, are both higher than 200, which in another perspective supports the idea that higher success rate of **Strategy 2** could be the result of considering higher ranked compounds due to the using of a lower number of compounds for clustering.

### Molecular diversity of VS candidates

The main purpose of clustering top-ranked 1,000 rather than fewer molecules in **Strategy 1** is to maximize the molecular diversity of the screened compounds. So we also compared the molecular diversity of the compounds from **Strategy 1** to that from **Strategy 2**. FCFP_6 fingerprint and tanimoto metric were used to calculate molecular diversity in DS 2.5. As is shown in Table 1, the molecular diversity of the candidates from both strategies was calculated based on the fingerprint distance metrics, which is defined as 1 - similarity for every pair of molecules. We can find that the average fingerprint distances for **Strategy 1** and **Strategy 2** are 0.881 and 0.859, respectively, which suggests that the molecular diversity of the candidates from **Strategy 2** with fewer clustering compounds still maintains compared with that from **Strategy 1**. The distributions of the fingerprint distance for the molecules from **Strategy 1** and **Strategy 2** were also plotted in Supplementary Fig. S4. We can find that both sets are normal-distributed and well overlapped. The maximum fingerprint distances are almost identical (**Strategy 1**: 0.959; **Strategy 2**: 0.958), but the minimum fingerprint distances are obviously different (**Strategy 1**: 0.621; **Strategy 2**: 0.370). This means that several compounds from **Strategy 2** are structurally similar. However these compounds have little influence on the average fingerprint distance, so most compounds from **Strategy 2** are still structurally diverse. Therefore, using fewer molecules in clustering analysis is suggested to improve the hit rates, but the exact number of compounds should be assessed in advance based on the molecular diversity of the selected compounds. However, the outcome from this study can only be a welcome indication when no extensive testing of the other protein targets is carried out. So this work can only be regarded as a case study, which is possibly target dependent. Several VS projects against other protein targets are ongoing in our lab, and we hope the outcome could be generalized.

### Biological activity and binding mechanisms of the active compounds

Based on the results of preliminary screening assays, ~10% compounds from **Strategy 1** and ~40% compounds from **Strategy 2** were found to inhibit ≥80% Tie-2 enzymatic activity. The dose-response effects of these candidate compounds were then evaluated to determine the half-maximal inhibitory concentrations (IC_50_). The compounds were tested in duplicate or more for every compound concentration. The dose-response curve was generated and plotted for each compound, and the IC_50_ value was calculated from each curve. The cutoff percentage inhibitory values of the compounds for IC_50_ determination were set to 60% and 80% for **Strategy 1** and **Strategy 2**, respectively. 6 compounds from **Strategy 1** and 18 compounds from **Strategy 2** were finally tested, and the results were summarized in Table 2. The IC_50_ values of 13 compounds from **Strategy 2** (TP-C2) were under 10 μM, while only 2 compounds from **Strategy 1** (TP-C1) exhibited <10 μM activity.

**Figure 5:**
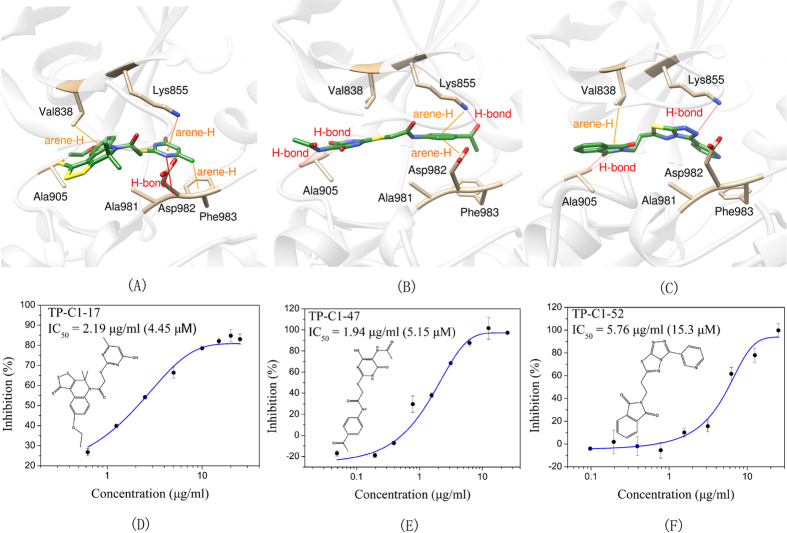
Schematic representations of the binding modes of compounds (**A**) TP-C1-17, (**B**) TP-C1-47 and (**C**) TP-C1-52, and dose-dependent inhibitory effects (IC_50_ values) of compounds (**D**) TP-C1-17, (**E**) TP-C1-47 and (**F**) TP-C1-52.

**Figure 6:**
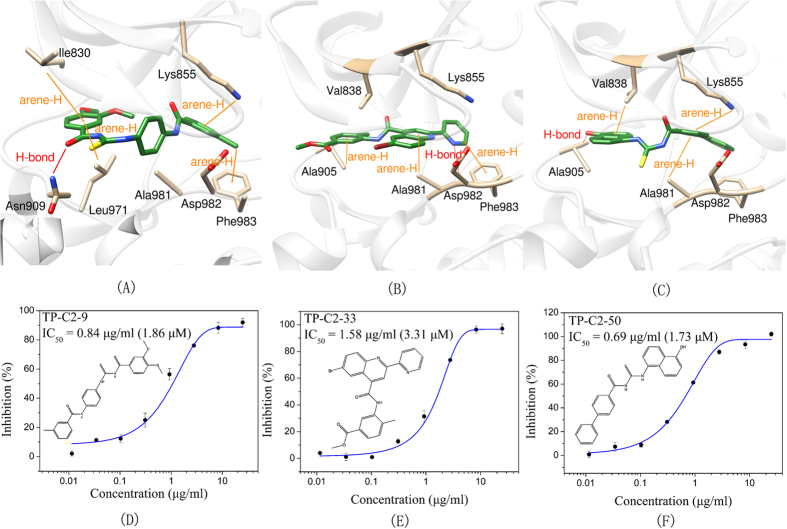
Schematic representations of the binding modes of compounds (**A**) TP-C2-9, (**B**) TP-C2-33 and (**C**) TP-C2-50, and dose-dependent inhibitory effects (IC_50_ values) of compounds (**D**) TP-C2-9, (E) TP-C2-33 and (**F**) TP-C2-50.

The IC_50_ values of three most potent compounds from each strategy (TP-C1-17, 47, 52 and TP-C2-9, 33, 50) are 4.45 μM, 5.15 μM, 15.3 μM, 1.86 μM, 3.31 μM, and 1.73 μM, respectively (Figs 5 and 6). The novelty of these inhibitors was also assessed by evaluating the structural similarity to the reported type-I inhibitors of Tie-2 that were retrieved from the BindingDB database. The molecular similarity was computed based on the pairwise Tanimoto similarity using FCFP_6 fingerprints^41^. According to our calculations, the maximum similarities of compounds TP-C1-17, 47, 52 and TP-C2-9, 33, 50 are 0.125, 0.141, 0.147, 0.268, 0.204, and 0.252, respectively, where all the obtained known inhibitors were compared to each compound and the similarity value of the most similar inhibitor was kept. All the 6 compounds show low structural similarity to the reported inhibitors, indicating that the candidate compounds discovered are novel Tie-2 inhibitors.

The binding mechanisms of three most potent inhibitors from each strategy were further investigated by the Glide XP docking simulations (Figs 5 and 6). The dominating residues for the binding of these 6 compounds are Val838, Lys855, Ala905, Ala981, Asp982 and Phe983. However, the interaction pattern in each inhibitor/Tie-2 system is different. In the binding of compound TP-C1-17 to Tie-2, the nitrogen atom of pyrazine group in TP-C1-17 can form a hydrogen bond with the backbone of Asp982. The pyrazine ring and the substituted methyl group can form arene-H contacts with the residues Lys855 and Phe983. In addition, arene-H interaction can also be found between the benzene ring and the residue Val838. The inhibitory activity of the compound TP-C1-47 is similar to that of TP-C1-17, but the interaction patterns are relatively different. Three hydrogen bonds interacting with the residues Lys855 and Ala905 are observed. The side chains of Asp982 can form arene-H interactions with the benzene ring in TP-C1-47 rather than a hydrogen bond. The role of Lys855, which interacts with the benzene ring also via arene-H contacts, is similar to the binding of TP-C1-17. The activity of compound TP-C1-52 is ~3 fold decreased compared to those of TP-C1-17 and TP-C1-47. This may result from the lack of strong hydrophobic interactions in the surrounding region of Asp982, Phe983 and Lys855, where only a hydrogen bond is observed between Lys855 and the nitrogen atom of the thiadiazole group. Another hydrogen bond can also be found between the carbonyl of isoindole and the backbone of Ala905. In addition, the isoindole ring in TP-C1-52 can also form arene-H interactions with Val838.

In the binding of compound TP-C2-9, the benzene rings in both sides of the molecules, which can form π interactions with Lys855, Phe983, Ile830 and Leu971, play major roles in ligand binding. Different from the other 5 compounds, TP-C2-9 can form a hydrogen bond with Asn909 showing a unique binding mode of action. For compound TP-C2-33, hydrophobic interactions also dominate the binding to Tie-2, where Ala905, Ala981 and Phe983 form arene-H interactions with TP-C2-33. A hydrogen bond is also formed between the nitrogen atom of pyridine group in TP-C2-33 and the backbone of Asp982, which is similar to the hydrogen binding of TP-C1-17. In the structure of TP-C2-50, the biphenyl group and naphthalene ring are quite hydrophobic, which form strong arene-H interactions with Val838, Lys855 and Ala981. Besides, the substituted hydroxyl group in naphthalene ring can interact with the backbone of Ala905 via hydrogen bonding.

### Preliminary structure−activity relationship (SAR) analysis

The candidate compound (TP-C2-9), which represents novel chemotypes for Tie-2 inhibitors, was selected for further optimization and preliminary SAR analysis. Hit optimization was performed based on the technique of substructure search to retrieve the compounds that contain the query substructure in ChemBridge database. The core scaffold of compound TP-C2-9, which is colored red in Tables 3 and 4, was taken as the template structure for searching. 60 analogues of TP-C2-9 were finally selected and purchased for the second-round screening. These compounds were firstly assessed for their ability to inhibit Tie-2 at the concentration of 5 μg/mL. The results of kinase assay clearly revealed that different substitutes on R1, R2 and R3 phenyl ring have obvious effects on the inhibitory activity. The IC_50_ values of six compounds (TP-C3-1, TP-C3-3, TP-C3-21, TP-C3-43, TP-C3-45 and TP-C3-55) are lower than 5 μM, wherein the most potent one (TP-C3-43) can reach 1.64 μM. However, we can also observe that most analogues exhibit weak inhibitory activity (<50% at 5 μg/mL). The low hit rates of the compounds from substructure search, on the contrary, demonstrated the effectiveness of VS that selected TP-C2-9 (IC_50_ = 1.86 μM) rather than other analogues as the preferential compound.

**Figure 7:**
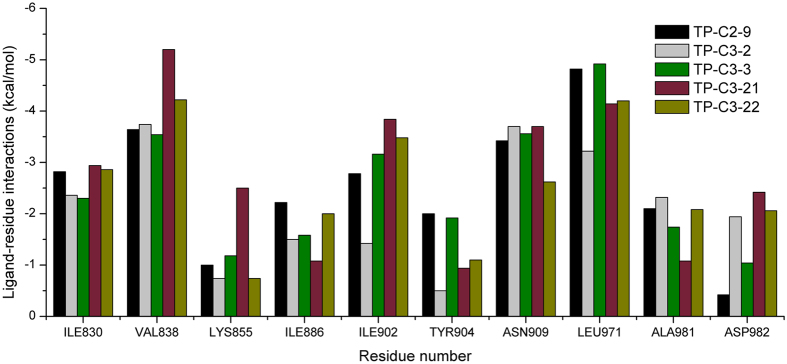
MM/GBSA decomposition analysis of the energetic contributions of the dominating amino acids on a per-residue basis for compounds TP-C2-9, TP-C3-2, TP-C3-3, TP-C3-21 and TP-C3-22. The diagram was generated based on the average structures from the last stable 3 ns conformations of each MD simulation.

The inhibitory activity of compound TP-C3-3 (IC_50_ = 2.82 μM) is only slightly decreased compared to that of TP-C2-9, where two methoxy groups are removed from the R2 phenyl ring. This indicates that the removal of methoxy groups has little influence on ligand binding. However, replacement of 3-methyl group by 4-tert-butyl in R1 ring (compound TP-C3-2) leads to~10 fold decreases in activity compared with compound TP-C3-3. To gain rational insights into the difference of the binding modes, MM/GBSA binding energy calculations and decomposition analysis based on MD simulations were performed by examining the contribution of each residue to binding. Figure 7 illustrated the energetic contributions of the dominating residues from the MM/GBSA decomposition analysis. We can find that the contributions of most residues for both compounds TP-C2-9 and TP-C3-3 are similar, implying that removal of methoxy groups from R2 ring does not affect the main interactions between inhibitors and Tie-2. For the binding of compound TP-C3-2 (see Supplementary Fig. S1), several critical interaction patterns are influenced, especially the arene-H contacts with the residues Ile902 and Leu971. As illustrated in Fig. 7, the contributions of Ile902 and Leu971 for TP-C3-2, which are dominated by the non-polar interactions, are obviously smaller than those for TP-C3-3. In addition, the impaired interactions with Tyr904 also account for the inferior binding affinity of TP-C3-2. Interestingly, the binding to Asp982, where arene-H interactions can be observed, is found stronger than both compounds TP-C2-9 and TP-C3-3, however, this cannot make up for the decreased binding affinity to other amino acids.

The methyl group on R1 was found crucial for inhibitor binding since the inhibitory activities of all the compounds (TP-C3-4~TP-C3-15 and TP-C3-26~TP-C3-30) were greatly reduced when R1 methyl is removed. The IC_50_ value of the most potent one within this series (TP-C3-15) is only 13.79 μM with ~10 fold decline in activity compared to TP-C2-9. This phenomenon is mainly caused by the interruption of arene-H contacts between Phe983 and methyl group. Substitutions of methyl groups on different positions of R2 phenyl can significantly influence the potency. Compound TP-C3-21 with methyl groups introduced on 2, 4-positions of R2 ring displays strong inhibitory activity (71.19% inhibition, 5 μg/mL), while almost no activity was observed for TP-C3-22 with methyl groups substituted on 3, 5-positions. Detailed energy decomposition analysis revealed that the impaired interactions with the residues Val838, Lys855 and Asn909 determinate the decreased binding affinity of TP-C3-22 to Tie-2. The residues Val838 and Lys855 can form arene-H contacts with compound TP-C3-21, but these interactions are not observed in the binding of TP-C3-22 (see Supplementary Fig. S1), which directly leads to the reduction of the binding energy. Except for the impaired van der Waals interactions with Val838 and Lys855, the electrostatic interactions with Asn909 (~1 kcal/mol drop in energy) are also responsible for the decreased binding affinity. Thus, introducing methyl groups on different positions of R2 phenyl ring can cause structural rearrangement of the inhibitor as well as the side chains of surrounding amino acids, which leads to different binding potency. Compound TP-C3-43 is the most potent inhibitor with an IC_50_ value of 1.64 μM, but replacement of chlorine by methyl on the 3-position of R1 ring (TP-C3-47) is unfavorable. Structural analysis (see Supplementary Fig. S1) revealed that most critical interactions are not observed in the binding of TP-C3-47 to Tie-2. Even small variations in structure may cause significant change of inhibitor-protein interaction patterns and finally lead to the change of activity. Compounds listed in Table 5 mainly focus on modifications with saturated or unsaturated six-member rings introduced on R1 or R2 phenyl, but no compounds displayed strong inhibitory activity at the concentration of 5 μg/mL, implying that no sufficient space around R1 or R2 ring can accommodate bulk groups.

Based on the discussions above, most of the dominating amino acids are hydrophobic, so the non-polar interactions, especially the bindings to the residues Ile830, Val838, Ile902, Ala981 and Leu971 via π/arene-H contacts, play key roles in inhibitor binding. In addition, the hydrophilic regions involving the residues Lys855, Asn909 and Asp982 may benefit to form H-bond interactions and should be paid special attention in further optimization. The inhibitors discovered and the detailed binding modes of action facilitate the subsequent focused medicinal chemistry study to expand the SAR of potential drug candidates.

## Conclusions

Targeted intervention of ANG/Tie-2 signaling pathway in preliminary vessels represents a promising strategy for the treatment of various life-threatening diseases. In the present work, a class of compounds was identified as potential inhibitors of Tie-2 through structure-based VS and substructure searching. By comparing the performance of two different filtering strategies employed in VS, we found that the hit rate can be well improved when stricter drug-likeness criteria and top-200 rather than top-1000 compounds for clustering analysis were used, and meanwhile, the molecular diversity of the compounds still maintained. Further hit-based optimization was also performed to give a preliminary SAR using substructure searching technology. The information presented and the novel inhibitors discovered in this work will benefit to developing new anti-angiogenesis drug candidates.

## Materials and Methods

### Preparations for Protein Structures and Small Molecule Datasets

All structure preparations for molecular docking were carried out using the *Protein Preparation wizard* in Maestro 9.0 program^42^ starting from previously reported crystal structures of human Tie-2 in complex with CEP11207 (PDB code: 3L8P)^21^ and a thiazolopyrimidine inhibitor (PDB code: 2OO8)^43^. All water molecules in the original crystal structures were removed from the coordinate set. Hydrogen atoms and partial charges were added to the protein, followed by a restrained partial minimization which is terminated when the root-mean-square deviation reached 0.3 Å.

The reported inhibitors of Tie-2 were retrieved from the BindingDB database^44^. In order to evaluate the prediction accuracy of docking simulation in distinguishing type-I inhibitors from non-inhibitors, the type-II inhibitors and those with high IC_50_ values (IC_50_ > 100 μM) were removed from the dataset of known inhibitors^45^. The eventual inhibitor dataset was composed of 200 structurally diverse molecules by extracting from the above cleared dataset using the *Find Diverse Molecule* module in Discovery Studio 2.5 (DS 2.5)^46^. The 20,000 non-inhibitors of Tie-2 was randomly selected from the ChemBridge chemical database (~1,020,000 screening compounds) using DS 2.5^46^. All the selected inhibitors and non-inhibitors were then minimized using the OPLS-2005 force field^47^. Preparations of all the compounds were subsequently performed using the *LigPrep* module in Schrödinger 9.0^48^, where the protonated states were predicted at pH = 7.0 ± 2.0.

### Molecular Docking Simulations

The *Glide* module in Schrödinger was utilized to perform all the docking simulations. The protein grid box for molecular docking was produced by centering on the ligand in the active site using the *Receptor gird generation* module. The scaling factor for van der Waals radii was set to 0.8, and the partial atomic charge cutoff value was set to 0.15. Eventually, all the prepared compounds were docked into the corresponding crystal structures of Tie-2. Two different precision modes of docking were utilized, including the standard precision (SP) and extra precision (XP). Though the computational speed of the high throughput virtual screening (HTVS) mode is the fastest among the three docking modes, the prediction accuracy is quite low^49^. So the HTVS docking mode was not employed in our VS workflow.

### Molecular Dynamics (MD) Simulations and Binding energy Calculations

MD simulations were carried out to elucidate the binding patterns of the studied inhibitors. The Tie-2/inhibitor complexes predicted by the docking simulations were employed as the starting structures for the MD simulations. All the MD simulations were carried out in Amber14^50^. The electrostatic potential of each inhibitor was calculated at the HF/6-31 G* level after optimization by semi-empirical AM1 method in Gaussian 09 program^51^. Then, the atomic partial charges of the inhibitors were generated by fitting the electrostatic potentials using the RESP fitting method^52^.

The ff99SB force field^53^ and the general AMBER force field (*gaff*)^54^ were used for the proteins and inhibitors, respectively. Each system was inserted into a cubic TIP3P water box with at least 8 Å away from any solute atom. The long-range electrostatics was handled by the Particle Mesh Ewald (PME) method^55^.

Each system was firstly minimized by the *sander* program in three steps: 1) 500 cycles of steepest descent and 500 cycles of conjugate gradient minimizations with 50 kcal/mol/Å^2^ restraint on protein backbone; 2) 500 cycles of steepest descent and 500 cycles of conjugate gradient minimizations with 10 kcal/mol/Å^2^ restraint; 3) 1000 cycles of steepest descent and 4000 cycles of conjugate gradient minimizations without any restraint. Afterwards the systems were gradually heated from 0 to 300 K in 50 ps with 2.0 kcal/mol/Å^2^ restraint, and 5 ns NPT (*P* = 1 atm and *T* = 300 K) MD simulations were then carried out with a time step of 2 fs. All hydrogen bonded atoms were constrained using the SHAKE algorithm^56^. The interval time of saving the coordinates was set to 10 ps.

The Molecular Mechanics/Generalized Born Solvent Area (MM/GBSA) method implemented in the *mm_pbsa* module in Amber11 was employed to calculate the binding energies of the studied inhibitors^57^,^58^,^59^,^60^,^61^. The dielectric constants for solvent and solute were set to 80 and 1, respectively. The modified GB model (*igb* = 2)^62^ developed by Onufriev and coworkers was used to calculate the polar part of desolvation energy, and the LCPO algorithm^63^ was employed to compute the non-polar part of desolvation based on the solvent accessible surface area (SASA): Δ*G*_SA_ = 0.0072 × ΔSASA. Due to the low prediction accuracy and relatively high computational cost, the entropic contributions to the total binding free energy were not calculated^57^,^64^. Eventually, 200 structures evenly extracted from 3 to 5 ns MD trajectories were used to predict binding energies.

The MM/GBSA decomposition analysis was used to highlight the important residues to ligand binding^59^,^65^. Except for the non-polar part of the solvation free energy (∆*G*_SA_), which was calculated based on ΔSASA using the ICOSA algorithm^65^, the other terms were calculated based on the same parameters used in the MM/GBSA calculations.

### Reagents and Materials for Experiments

All anhydrous solvents and reagents used in this work were obtained from commercial sources. The Tie-2 kinase inhibitor (CAS#: 948557-43-5, purity ≥ 97%) was purchased from Merck Calbiochem (Darmstadt, Germany) and used as a positive control inhibitor in bioassays^40^. Both the purified recombinant human Tie-2 protein (Catalog#: PV3628) and Z′-Lyte^®^ kinase assay kit (Catalog#: PV3178) were purchased from Thermo Fisher Scientific Inc. The candidate compounds from VS were purchased from ChemBridge and were tested by LC-MS and/or NMR to confirm sample identity and purity (compound information are listed in Supplementary Table S1). All the purchased screening compounds were firstly dissolved in dimethyl sulfoxide (DMSO) at a stock concentration of 5 mg/ml. The final working concentration of DMSO is less than 1%.

### Enzymatic Activity Assay

Compounds were tested for their ability to inhibit Tie-2 kinase activity using Z′-Lyte^®^ kinase assay kit (ser/thr 5 peptide) with NUNC 384-well plate. In the first step, the test compounds and ATP solution were firstly prepared at 4 × desired concentration. The mixture of recombinant human Tie-2 protein and non-phosphorylated peptide at 2 × desired concentration was prepared in kinase reaction buffer A consisting of 50 mM HEPES (pH 7.5), 0.01% BRIJ-35, 10 mM MgCl2, 4 mM MnCl2, 1 mM EGTA and 2 mM DTT. Reactions were then carried out in a solution containing 2.5 μL test compounds, 5 μL Tie-2/Tyr 05 peptide mixture (10 ng Tie-2 and 2 μM Tyr 05 peptide) and 2.5 μL ATP (100 μM). Then, the assay plate was shaken for 30 seconds on a plate shaker, and incubated for 1 hour at room temperature (20~25 °C). After sufficient kinase reactions, 5 μL development reagent A was added to each well, and the plate was incubated for another hour. Finally, 5 μL stop reagent was added to terminate the reactions, and the coumarin and fluorescein emission signals (excitation: 400 nm; emission: 445 and 520 nm, respectively) were measured on a fluorescence plate reader (BioTek Synergy 4).

## Additional Information

**How to cite this article**: Pan, P. *et al*. *In Silico* Exploration for Novel Type-I Inhibitors of Tie-2/TEK: The Performance of Different selection strategy in Selecting Virtual Screening Candidates. *Sci. Rep.* **6**, 37628; doi: 10.1038/srep37628 (2016).

**Publisher’s note:** Springer Nature remains neutral with regard to jurisdictional claims in published maps and institutional affiliations.

## Supplementary Material

Supplementary Materials

## Figures and Tables

**Table 1:** Molecular Diversity of the Candidates from Two Different Filtering Strategies Based on Fingerprint Distance Metrics^a^.

	Candidates from **Strategy 1**	Candidates from **Strategy 2**
Average Fingerprint Distance	0.881178	0.859663
Minimum Fingerprint Distance	0.621359	0.370370
Maximum Fingerprint Distance	0.959350	0.958333

^a^Fingerprint distance is defined as 1 - similarity for every pair of molecules.

**Table 2:** Half-Maximal Inhibitory Concentrations (IC_50_) of the Active Compounds from Two Filtering Strategies.

Compd	IC_50_, μM ± SD^a^	Compd	IC_50_, μM ± SD^a^	Compd	IC_50_, μM ± SD^a^
TP-C1-4	34.03 ± 1.62	TP-C2-5	13.79 ± 1.02	TP-C2-33	3.31 ± 0.23
TP-C1-17	4.45 ± 0.36	TP-C2-9	1.86 ± 0.18	TP-C2-34	6.55 ± 0.46
TP-C1-28	43.64 ± 2.32	TP-C2-11	15.12 ± 0.92	TP-C2-35	4.59 ± 0.29
TP-C1-37	36.12 ± 1.33	TP-C2-12	14.56 ± 1.21	TP-C2-37	10.75 ± 0.83
TP-C1-47	5.15 ± 0.40	TP-C2-13	6.78 ± 0.36	TP-C2-38	7.88 ± 0.35
TP-C1-52	15.30 ± 0.68	TP-C2-16	8.83 ± 0.58	TP-C2-40	4.69 ± 0.37
Ctrl inhibitor	0.31 ± 0.92	TP-C2-22	8.44 ± 0.93	TP-C2-41	6.12 ± 0.81
		TP-C2-24	17.53 ± 1.16	TP-C2-50	1.73 ± 0.17
		TP-C2-30	2.05 ± 0.11	TP-C2-55	8.21 ± 0.46

^a^All the inhibition values for every tested concentrations were the average of n ≥ 2 values. The IC_50_ values were calculated based on every dose response curves.

**Table 3:** Tie-2 Kinase Inhibitory Activity at 5 μg/mL and Half-Maximal Inhibitory Concentrations (IC_50_) of Compound TP-C3-1~TP-C3-30 Analogues.

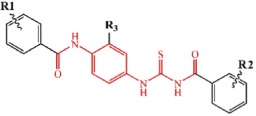					
Compd	R1	R2	R3	Kinase Inhibition (%) at 5 μg/mL ( ± SD)^a^	IC_50_, μM^a^
TP-C3-1	2,4-Cl	-H	-H	63.01 ± 0.86	1.85
TP-C3-2	4-C(CH_3_)_3_	-H	-H	33.36 ± 1.74	27.09
TP-C3-3	3-CH_3_	-H	-H	67.32 ± 0.78	2.82
TP-C3-4	-H	4-Cl	-H	12.84 ± 2.25	NT^b^
TP-C3-5	-H	4-NO_2_	-H	10.03 ± 0.85	NT^b^
TP-C3-6	-H	4-CH_3_	-H	45.24 ± 1.3	NT^b^
TP-C3-7	-H	4-OCH_3_	-H	43.12 ± 1.34	NT^b^
TP-C3-8	-H	4-O(CH_2_)_3_CH_3_	-H	26.08 ± 1.2	NT^b^
TP-C3-9	-H	4-O(CH_2_)_2_CH_3_	-H	16.16 ± 0.18	NT^b^
TP-C3-10	-H	3-Br, 4-OCH_2_CH_3_	-H	25.5 ± 1.79	NT^b^
TP-C3-11	-H	2-CH_3_	-H	4.66 ± 2	NT^b^
TP-C3-12	-H	2-Cl	-H	11.73 ± 1.66	NT^b^
TP-C3-13	-H	2-Br	-H	41.01 ± 0.8	NT^b^
TP-C3-14	-H	3-O(CH_2_)_2_CH_3_	-H	42.95 ± 1.08	22.66
TP-C3-15	-H	3-NO_2_, 4-OCH_3_	-H	35.09 ± 0.63	13.79
TP-C3-16	2-Cl	2-F	-OCH_3_	27.31 ± 1.46	NT^b^
TP-C3-17	2-Cl	4-C(CH_3_)_3_	-OCH_3_	45.22 ± 0.48	NT^b^
TP-C3-18	2-Cl	4-O(CH_2_)_3_CH_3_	-OCH_3_	11.07 ± 0.41	NT^b^
TP-C3-19	2-Cl	3-NO_2_, 4-OCH_3_	-OCH_3_	23.27 ± 0.16	NT^b^
TP-C3-20	2-Cl	3-OCH_2_-phenyl	-OCH_3_	10.41 ± 0.59	NT^b^
TP-C3-21	2-Cl	2-CH_3_, 4-CH_3_	-OCH_3_	71.19 ± 0.29	2.74
TP-C3-22	2-Cl	3-CH_3_, 5-CH_3_	-OCH_3_	6.42 ± 0.4	NT^b^
TP-C3-23	2-Cl	2-OCH_3_, 3-CH_3_, 5-Br	-OCH_3_	15.51 ± 0.11	NT^b^
TP-C3-24	2-Cl	2-CH_3_	-OCH_3_	18.49 ± 0.05	NT^b^
TP-C3-25	2-Cl	-H	-OCH_3_	14.14 ± 0.29	NT^b^
TP-C3-26	-H	3-NO_2_, 4-CH_3_	-CH_3_	23.11 ± 0.13	48.62
TP-C3-27	-H	2-OCH_3_, 5-Cl	-CH_3_	47.9 ± 0.05	19.75
TP-C3-28	-H	2-NO_2_	-CH_3_	4.09 ± 2.86	NT^b^
TP-C3-29	-H	3-Cl, 4-Cl	-CH_3_	57.95 ± 0.29	28.17
TP-C3-30	-H	4-OCH(CH_3_)CH_2_CH_3_	-CH_3_	17.52 ± 0.66	NT^b^
Positive Ctrl				93.01 ± 1.02	0.31

^a^All the inhibition values for every tested concentrations were the average of n ≥ 2 values. The IC_50_ values were calculated based on every dose response curves.

^b^NT, not tested.

**Table 4:** Tie-2 Kinase Inhibitory Activity at 5 μg/mL and Half-Maximal Inhibitory Concentrations (IC_50_) of Compound TP-C3-31~TP-C3-55 Analogues.

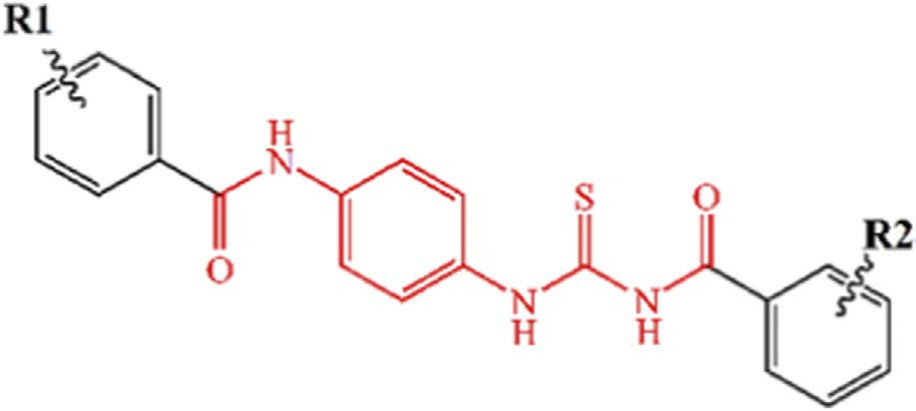				
Compd	R1	R2	Kinase Inhibition (%) at 5 μg/mL (±SD)^a^	IC_50_, μM^a^
TP-C3-31	3-Cl	4-Cl	25.8 ± 0.47	NT^b^
TP-C3-32	3-Cl	2-Cl	31.92 ± 1.57	NT^b^
TP-C3-33	3-Cl	3-Cl	8.61 ± 1.8	NT^b^
TP-C3-34	3-Cl	4-CH_3_	13.66 ± 1.61	NT^b^
TP-C3-35	3-Cl	3-Cl, 4-OCH_3_	11.13 ± 1.14	NT^b^
TP-C3-36	3-Cl	2-Br	24.24 ± 0.51	NT^b^
TP-C3-37	3-Cl	3-O(CH_2_)_2_CH_3_	33.66 ± 1.43	NT^b^
TP-C3-38	3-Cl	3-NO_2_, 4-OCH_2_CH_3_	31.21 ± 0.79	NT^b^
TP-C3-39	3-Cl	4-phenyl	−4.38 ± 0.98	NT^b^
TP-C3-40	3-Cl	2-Cl, 3-Cl	38.61 ± 0.21	NT^b^
TP-C3-41	3-Cl	2-Cl, 4-NO_2_	19.81 ± 0.09	NT^b^
TP-C3-42	3-Cl	2-Cl, 5-Cl	6.74 ± 0.5	NT^b^
TP-C3-43	3-Cl	4-CH_2_CH_3_	77.74 ± 0.42	1.64
TP-C3-44	3-CH_3_	4-Cl	17.74 ± 2.56	NT^b^
TP-C3-45	3-CH_3_	2-Cl	49.31 ± 0.95	2.41
TP-C3-46	3-CH_3_	2-CH_3_, 4-CH_3_	33.82 ± 0.1	NT^b^
TP-C3-47	3-CH_3_	4-CH_2_CH_3_	6.44 ± 1.06	NT^b^
TP-C3-48	4-Br	4-Cl	3.63 ± 1.8	NT^b^
TP-C3-49	4-Br	3-Cl	22.97 ± 0.18	NT^b^
TP-C3-50	4-C(CH_3_)_3_	4-CH_3_	23.81 ± 1.46	NT^b^
TP-C3-51	4-C(CH_3_)_3_	2-OCH_3_, 5-Cl	20.74 ± 0.55	NT^b^
TP-C3-52	4-C(CH_3_)_3_	4-O(CH_2_)_2_CH_3_	7.88 ± 0.59	36.74
TP-C3-53	4-C(CH_3_)_3_	2-OCH_3_, 6-OCH_3_	6.28 ± 0.19	NT^b^
TP-C3-54	4-C(CH_3_)_3_	3-CH_3_, 5-CH_3_	35.21 ± 0.01	NT^b^
TP-C3-55	2-Cl, 4-Cl	4-CH_2_CH_3_	60.22 ± 0.55	3.47
Positive Ctrl			93.01 ± 1.02	0.31

^a^All the inhibition values for every tested concentrations were the average of n ≥ 2 values. The IC_50_ values were calculated based on every dose response curves.

^b^NT, not tested.

**Table 5:** Tie-2 Kinase Inhibitory Activity at 5 μg/mL and Half-Maximal Inhibitory Concentrations (IC_50_) of Compound TP-C3-56~TP-C3-60 Analogues.

Compd	Structure	Kinase Inhibition (%) at 5 μg/mL (±SD)^a^	IC_50_, μM^a^
TP-C3-56	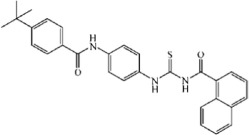	18.22 ± 1.31	NT^b^
TP-C3-57	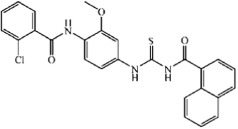	37.97 ± 0.43	NT^b^
TP-C3-58	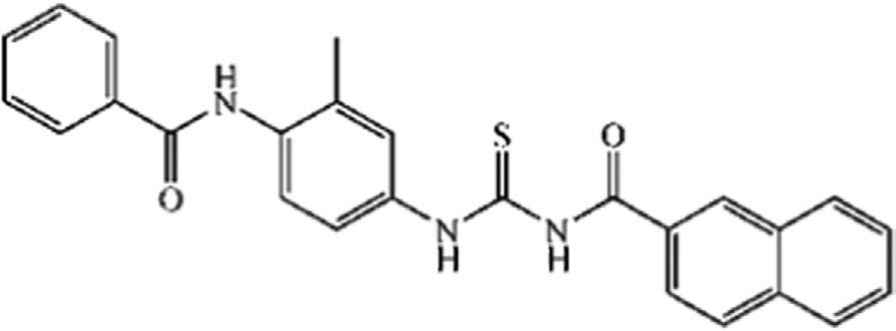	5.26 ± 0.51	NT^b^
TP-C3-59	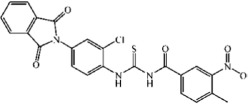	40.13 ± 0.39	NT^b^
TP-C3-60	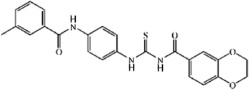	17.14 ± 0.31	NT^b^
Positive Ctrl		93.01 ± 1.02	0.31

^a^All the inhibition values for every tested concentrations were the average of n ≥ 2 values. The IC_50_ values were calculated based on every dose response curves.

^b^NT, not tested.
