# Supplementary material for: In Silico Exploration for Novel Type-I Inhibitors of Tie-2/TEK: The Performance of Different Selection Strategy in Selecting Virtual Screening Candidates
Source: Sci Rep. 2016 Nov 23;6:37628. doi: 10.1038/srep37628 (PMC5120310; doi:10.1038/srep37628)
Supplement: Supplementary Materials [file srep37628-s1.pdf]

Supplementary Information

In Silico Exploration for Novel Type-I Inhibitors of TIE-2/TEK: The Performance of Different Filtering Criteria in Selecting Virtual Screening Candidates

Peichen Pan[a], Huiyong Sun[a], Hui Liu[a], Dan Li[a], Wenfang Zhou[a], Xiaotian Kong[c], Youyong Li[c], Huidong Yu[d], Tingjun Hou[a,b]

[a]College of Pharmaceutical Sciences, Zhejiang University, Hangzhou, Zhejiang 310058, China

[b]State Key Lab of CAD&CG, Zhejiang University, Hangzhou, Zhejiang 310058, China

[c]Institute of Functional Nano and Soft Materials (FUNSOM), Soochow University, Suzhou, Jiangsu 215123, China

[d]Rongene Pharma Co., Ltd., International Business Incubator, Guangzhou Science Town, Guangdong 510663, China

Corresponding authors:

Tingjun Hou

E-mail: tingjunhou@zju.edu.cn or tingjunhou@hotmail.com

Table S1. Chemical information and structures of the VS candidates from ChemBridge database.

Compd	ID Number	Mol Weight	Inhibition (%) @10 µg/ml	Structure
Tie-2 inhibitor		439.5	95.2 ±1.58	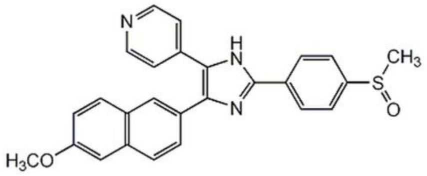
TP-C1-1	5100302	382.4634	20.58 ±5.19	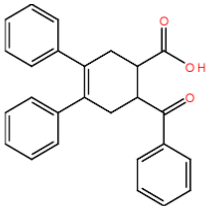
TP-C1-2	5169184	354.415	56.84 ±0.18	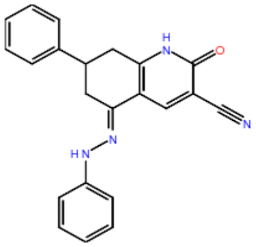
TP-C1-3	5237422	352.3498	37.64 ±1.82	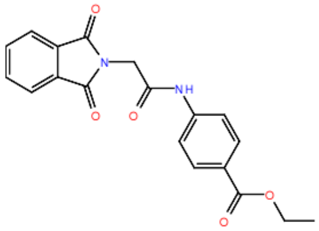
TP-C1-4	5381327	367.8599	43.36 ±1.3	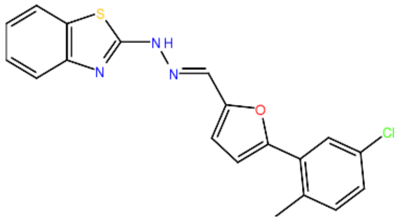
TP-C1-5	5653661	409.4513	25.58 ±3.23	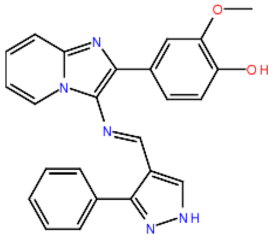

TP-C1-6	5662036	445.5284	16.08±16.2	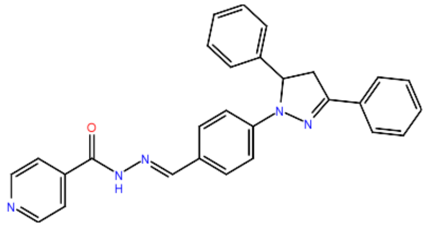
TP-C1-7	5748828	400.438	9.84±11.04	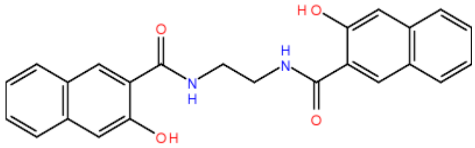
TP-C1-8	5793914	464.473	31.87±0.78	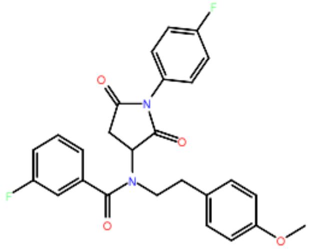
TP-C1-9	5929651	403.5259	17.49±0.65	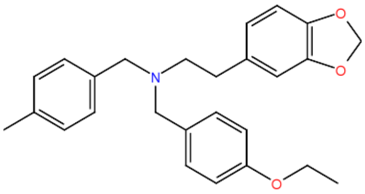
TP-C1-10	6054022	409.4485	44.25±1.05	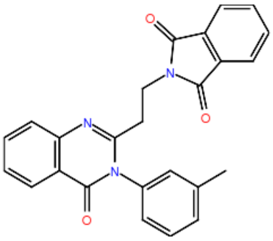
TP-C1-11	6147887	415.4278	37.56±1.85	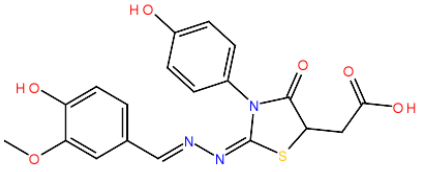
TP-C1-12	6321240	355.3968	3.6±13.1	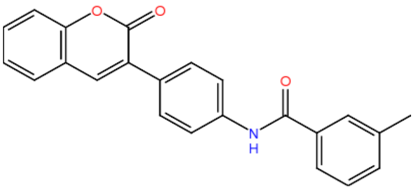

TP-C1-13	6431453	395.4622	28.98 ±1.34	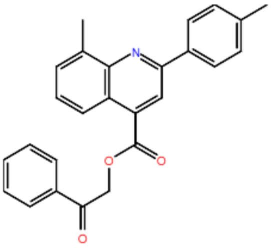
TP-C1-14	6463523	450.5361	10.67 ±4.7	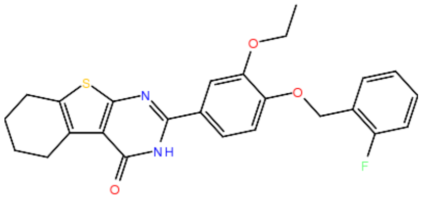
TP-C1-15	6498695	441.5125	-0.66 ±5.93	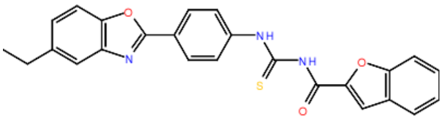
TP-C1-16	6594911	490.5855	9.52 ±4.54	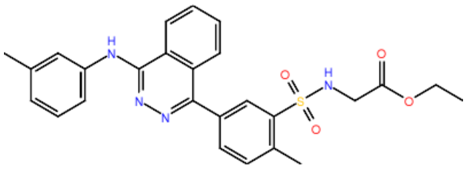
TP-C1-17	6599472	491.6758	89.54 ±2.93	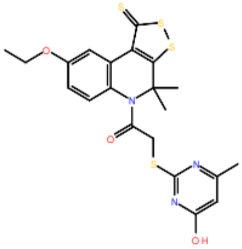
TP-C1-18	6628596	396.3694	39.7 ±2.94	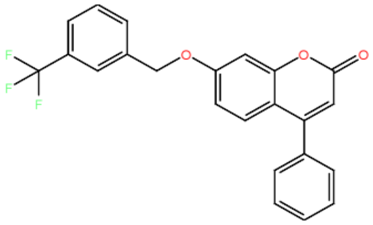
TP-C1-19	6633445	426.5387	30.01 ±7.54	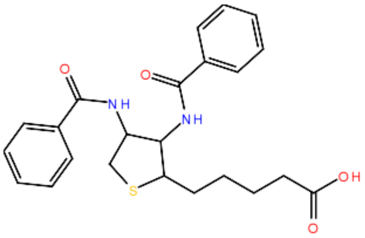

TP-C1-20	6636291	461.5686	43.21 ±0.45	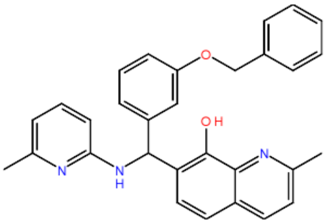
TP-C1-21	6657296	367.4733	58.96 ±3.25	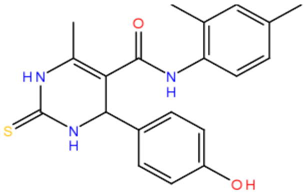
TP-C1-22	6675380	452.5084	-46.75 ±1.97	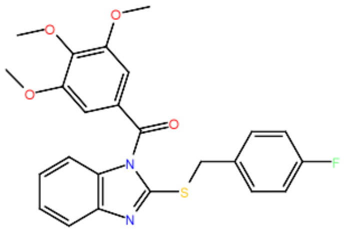
TP-C1-23	6688685	358.8001	8.02 ±9.13	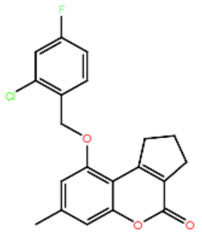
TP-C1-24	6770846	522.027	18.25 ±2.06	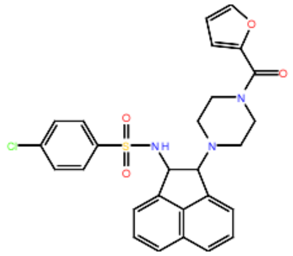
TP-C1-25	6881070	384.4358	3.13 ±1.45	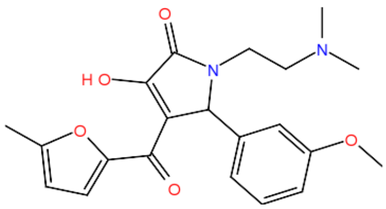
TP-C1-26	6893831	401.8503	7.19 ±0.3	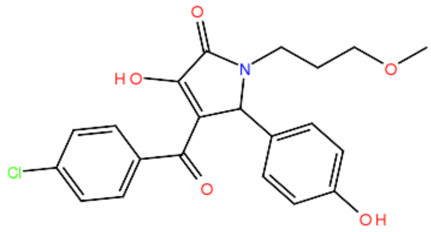

TP-C1-27	6932958	447.9193	4.93±5.56	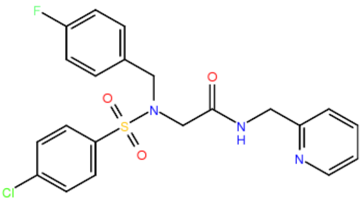
TP-C1-28	6944922	390.4423	71.3±0.7	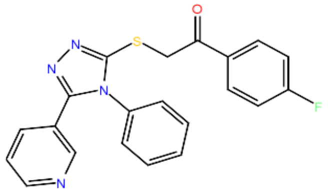
TP-C1-29	6990862	458.5839	39.83±5.94	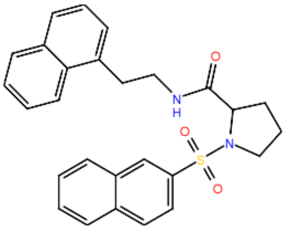
TP-C1-30	7055904	316.3627	11.44±0.49	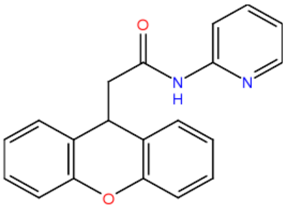
TP-C1-31	7114751	464.6317	2.9±8.41	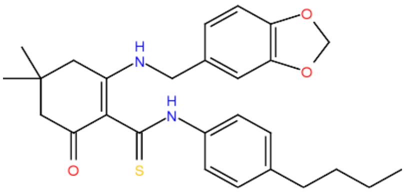
TP-C1-32	7221438	477.6084	47.23±0.53	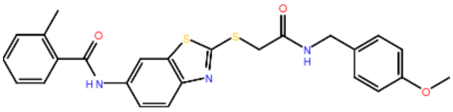
TP-C1-33	7384374	412.4867	18.01±0.97	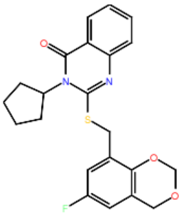
TP-C1-34	7480493	408.4145	6.95±6.26	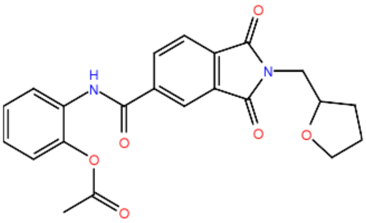

TP-C1-35	7583552	440.5249	69.07±3.54	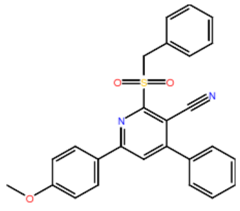
TP-C1-36	7636954	459.5715	3.87±1.8	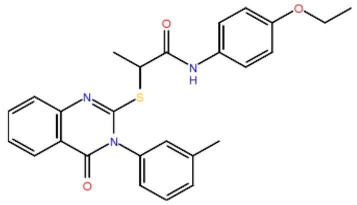
TP-C1-37	7731094	355.3997	25.51±1.18	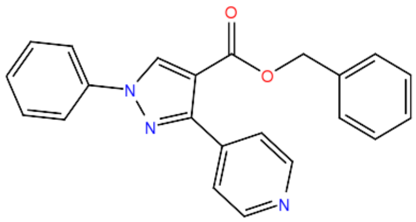
TP-C1-38	7743069	425.4667	12.5±2.86	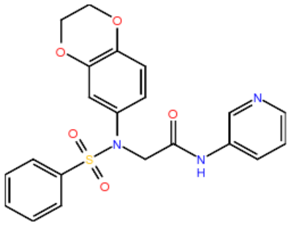
TP-C1-39	7753758	409.5512	56.09±2.14	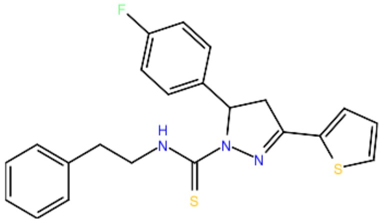
TP-C1-40	7783655	346.3643	2.81±1.73	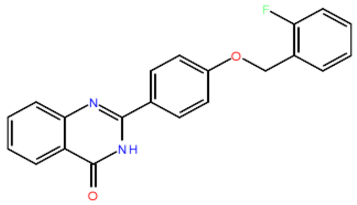
TP-C1-41	7826974	436.5804	32.86±3.03	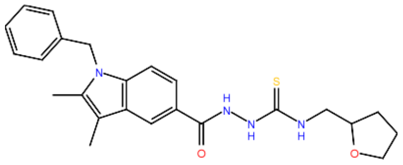
TP-C1-42	7853558	399.4068	-27.08±4.67	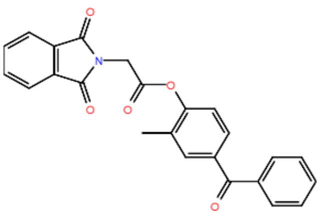

TP-C1-43	7917761	439.9241	-0.28 ±1.12	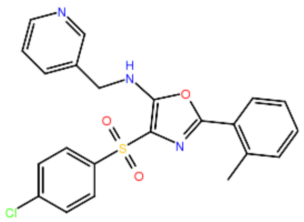
TP-C1-44	7114751	464.6317	18.69 ±6.98	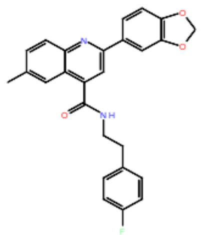
TP-C1-45	7923553	531.4134	63.88 ±1.45	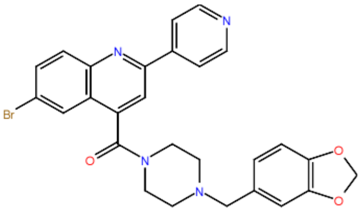
TP-C1-46	7963573	345.7855	18.81 ±0.06	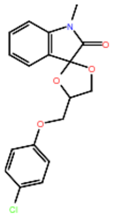
TP-C1-47	7964807	376.3937	81.92 ±1.73	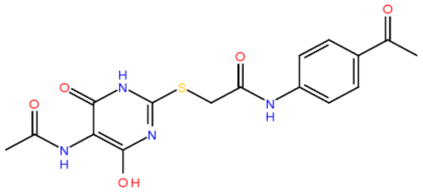
TP-C1-48	7968839	327.3891	34.8 ±0.04	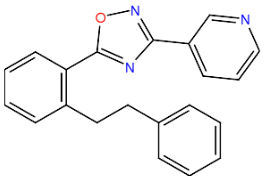
TP-C1-49	7968924	459.9117	49.58 ±1.56	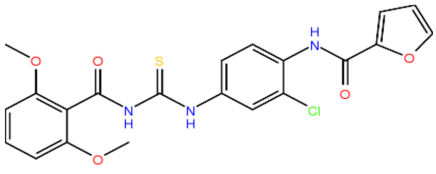
TP-C1-50	7970074	331.4018	61.04 ±3.33	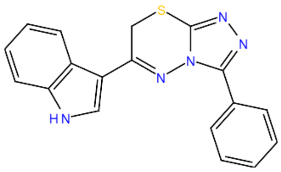

TP-C1-51	9011370	388.3832	54.56 ±1.99	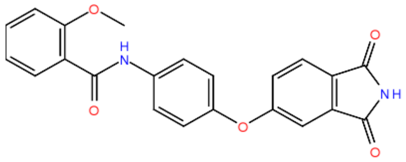
TP-C1-52	9017301	376.3993	100.9 ±2.04	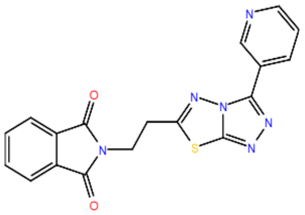
TP-C1-53	9026043	336.3776	28.39 ±0.73	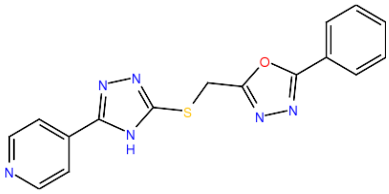
TP-C1-54	9084068	383.454	22.1 ±0.83	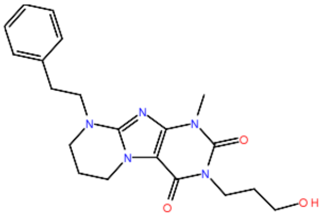
TP-C1-55	9131561	408.2847	20.01 ±0.68	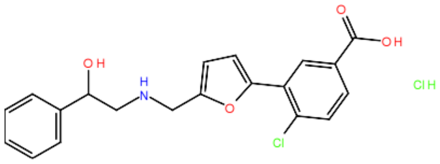
TP-C1-56	9154593	349.4392	29.83 ±0.28	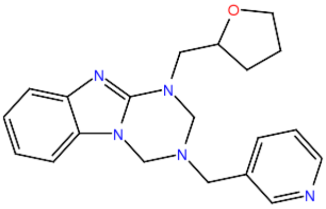
TP-C2-1	5579936	318.336	48.48 ±7.49	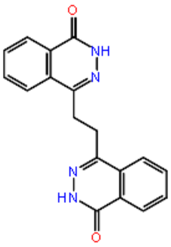

TP-C2-2	5648029	389.412	51.41 ±2.9	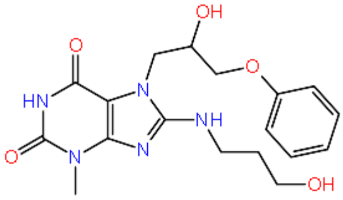
TP-C2-3	5772036	398.89	56.11 ±13.15	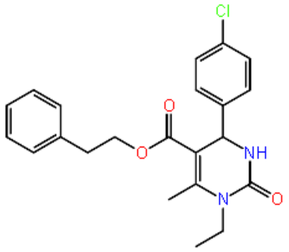
TP-C2-4	5787759	447.531	69.77 ±2.75	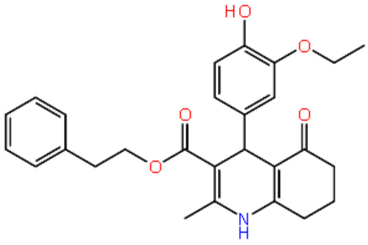
TP-C2-5	5793761	504.558	83.65 ±2.57	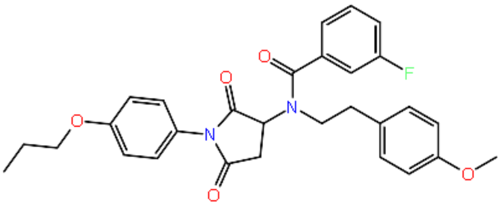
TP-C2-6	5928608	457.492	49.69 ±0.95	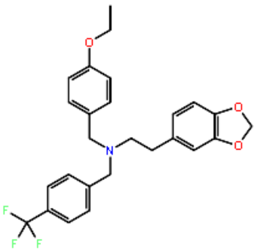
TP-C2-7	6382175	403.506	54.53 ±0.58	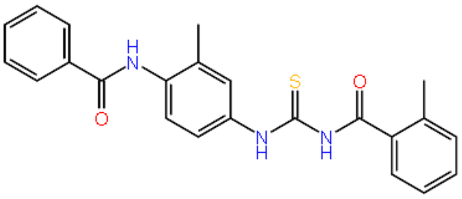
TP-C2-8	6382216	461.542	68.53 ±6.25	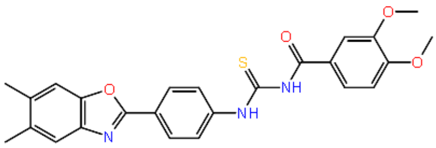

TP-C2-9	6391365	449.531	81.6±2.85	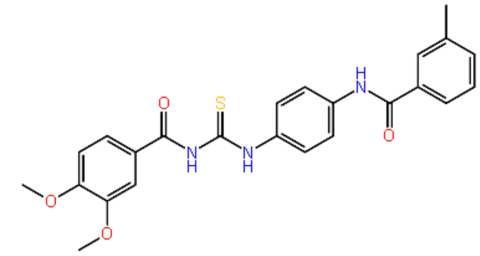
TP-C2-10	6394190	468.331	46.26±3.21	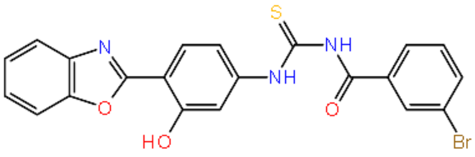
TP-C2-11	6473072	475.525	87.61±5.43	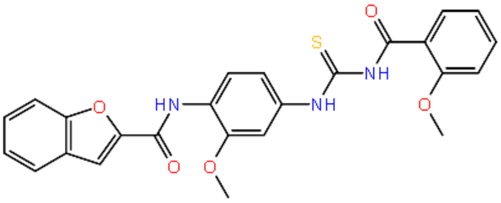
TP-C2-12	6503885	441.511	85.54±0.14	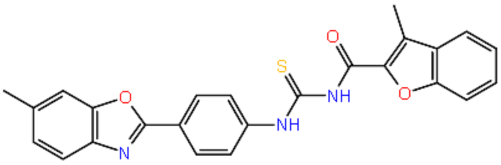
TP-C2-13	6546420	408.522	84.95±3.24	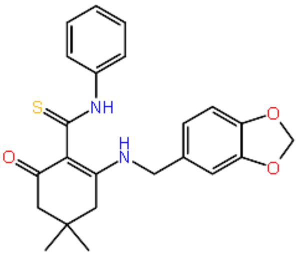
TP-C2-14	6548893	431.516	71.64±2.92	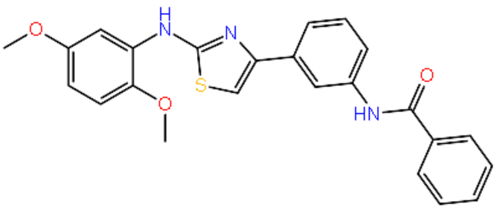
TP-C2-15	6552249	431.54	72.64±14.95	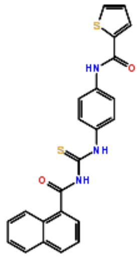

TP-C2-16	6561692	443.551	84.1 ±0.44	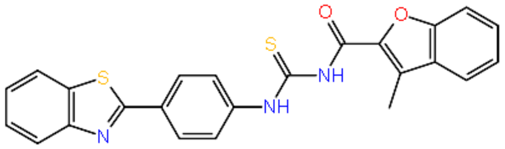
TP-C2-17	6683000	416.498	66.32 ±3.22	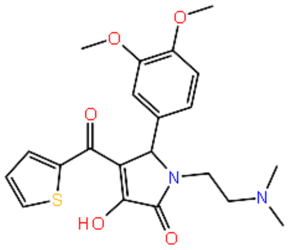
TP-C2-18	6687446	402.421	43.81 ±7.48	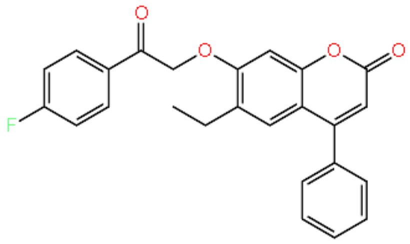
TP-C2-19	6689007	447.471	-280.42 ±23.11	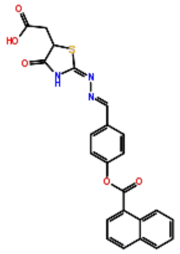
TP-C2-20	6767627	390.435	36.45 ±0.94	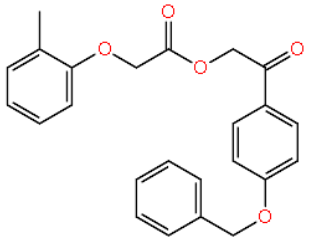
TP-C2-21	7332477	376.795	75.19 ±7.09	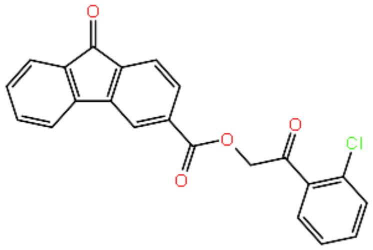
TP-C2-22	7559681	285.411	90.2 ±6.92	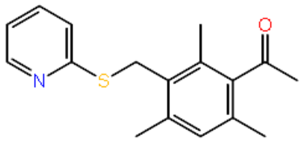

TP-C2-23	7650729	388.463	47.13±8.4	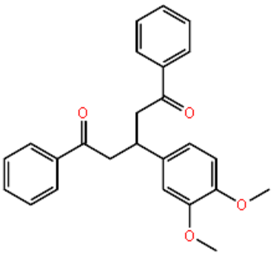
TP-C2-24	7677910	447.583	83.25±0.08	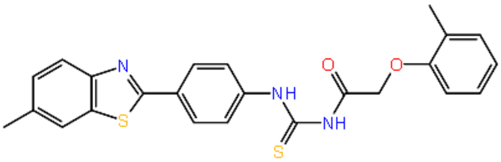
TP-C2-25	7758457	367.429	-113.04±17.27	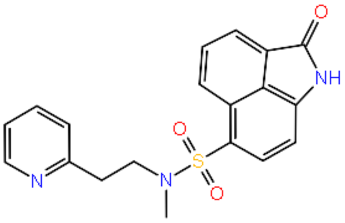
TP-C2-26	7780462	372.38	61.34±20.45	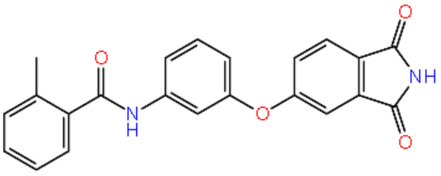
TP-C2-27	7802447	389.451	55.28±4.04	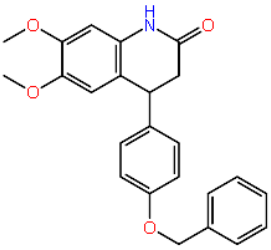
TP-C2-28	7818207	443.463	40.32±14.48	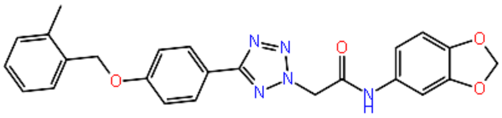
TP-C2-29	7840970	465.574	55.92±5.4	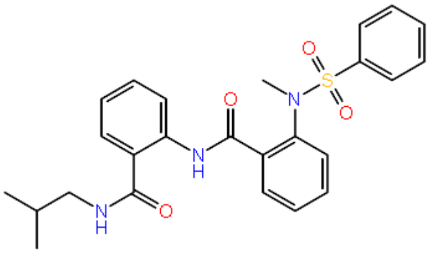

TP-C2-30	7854362	433.488	81.26±3.96	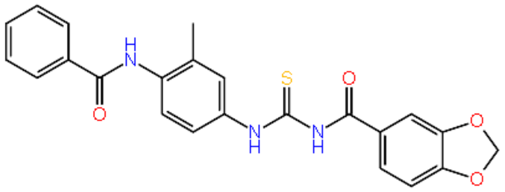
TP-C2-31	7912501	372.38	51.93±0.22	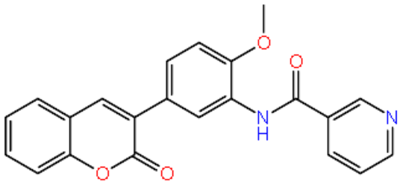
TP-C2-32	7921005	347.33	66.52±7.26	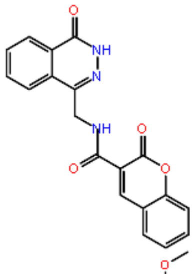
TP-C2-33	7921999	476.33	95.17±1.81	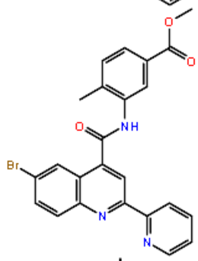
TP-C2-34	7922196	522.029	80.32±0.86	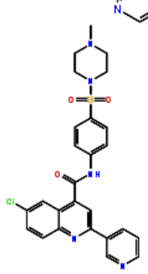
TP-C2-35	7922533	363.804	87.59±3.7	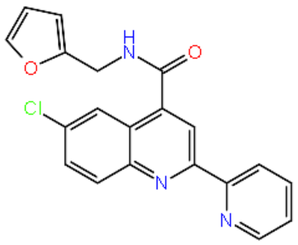
TP-C2-36	7922706	450.926	61.48±4.57	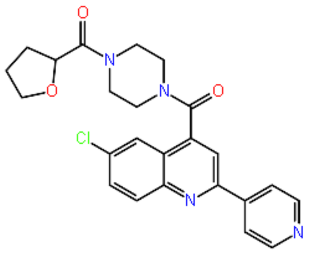

TP-C2-37	7923291	393.49	80.89±0.31	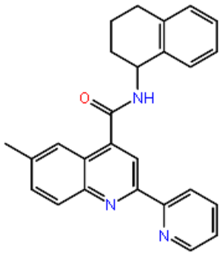
TP-C2-38	7923841	466.949	89.98±1.62	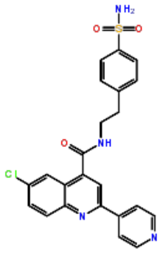
TP-C2-39	7923852	461.319	75.1±0.83	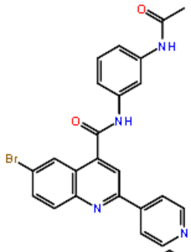
TP-C2-40	7930624	457.598	95.39±0.98	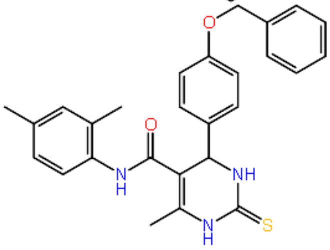
TP-C2-41	7931662	396.427	90.36±1.3	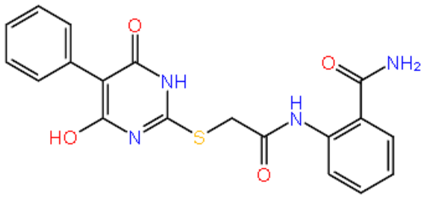
TP-C2-42	7932718	477.025	23.47±2.17	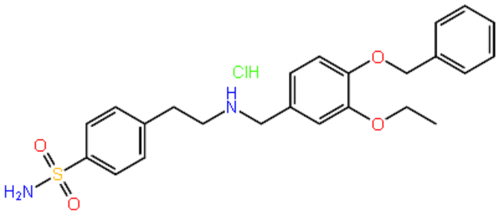
TP-C2-43	7951734	315.353	55.24±1.85	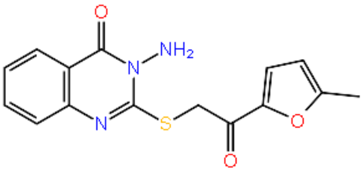

TP-C2-44	7952343	309.325	52.94 ±2.54	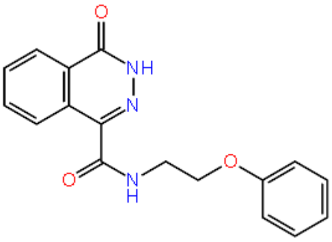
TP-C2-45	7954917	448.547	65.92 ±4.36	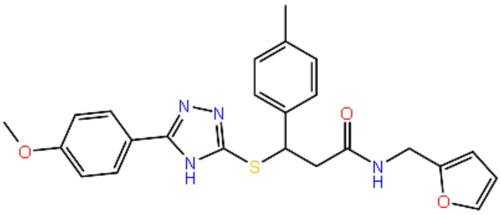
TP-C2-46	7976441	420.896	76.43 ±1.16	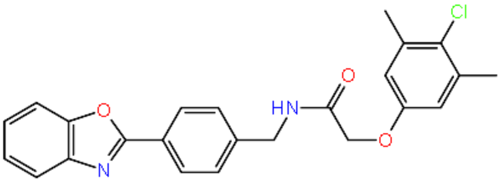
TP-C2-47	7977365	429.5	67.14 ±5.68	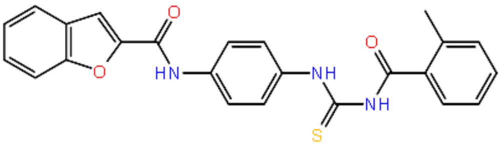
TP-C2-48	7980213	366.42	43.96 ±2.34	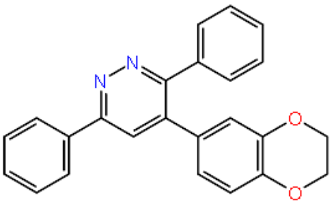
TP-C2-49	7986007	444.555	63.31 ±0.29	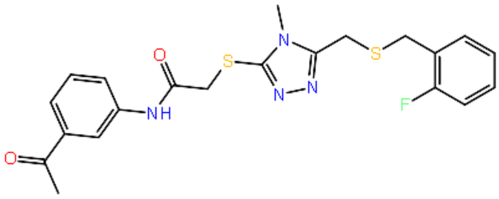
TP-C2-50	7986469	398.486	97.82 ±1.13	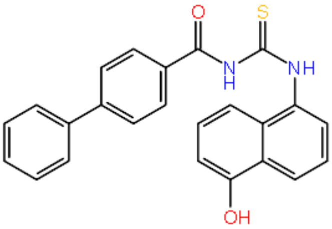

TP-C2-51	9010722	295.298	77.63 ±0.16	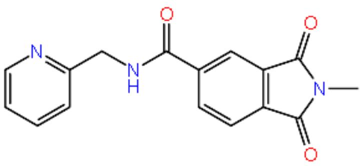
TP-C2-52	9031850	423.493	64.42 ±0.23	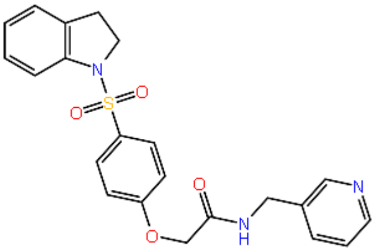
TP-C2-53	9059770	429.472	77.43 ±0.89	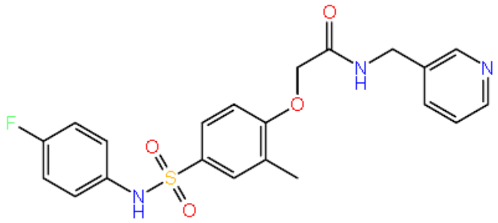
TP-C2-54	9111170	326.359	60.19 ±2.33	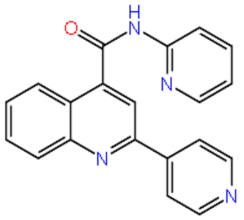
TP-C2-55	9118652	349.365	81.27 ±3.01	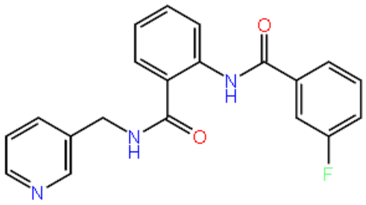
TP-C2-56	9133308	409.489	74.86 ±1.02	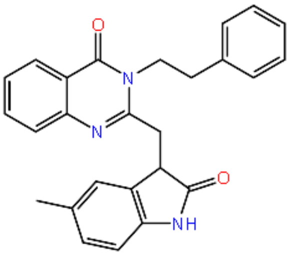
TP-C2-57	9148495	423.444	48.64 ±1.91	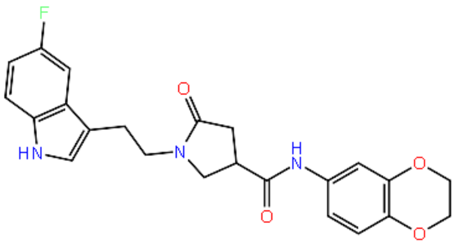

TP-C2-58	9158078	380.428	50.65 ±0.04	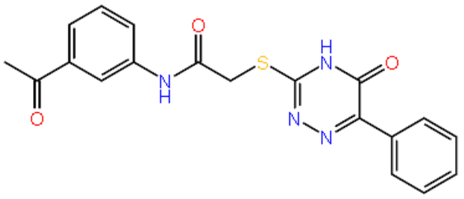
TP-C2-59	9188714	365.389	74.19 ±1.14	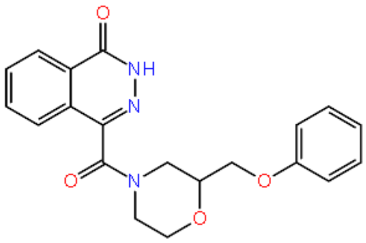
TP-C2-60	9190647	310.381	66.88 ±8.04	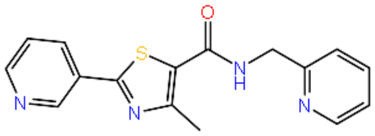

Table S2. Power(s) for Hypothetical Sample Size(s)

Alpha	Sample Size	Power
0.05	10	0.323607
0.05	20	0.589348
0.05	50	0.93956
0.05	100	0.998779
0.05	200	1

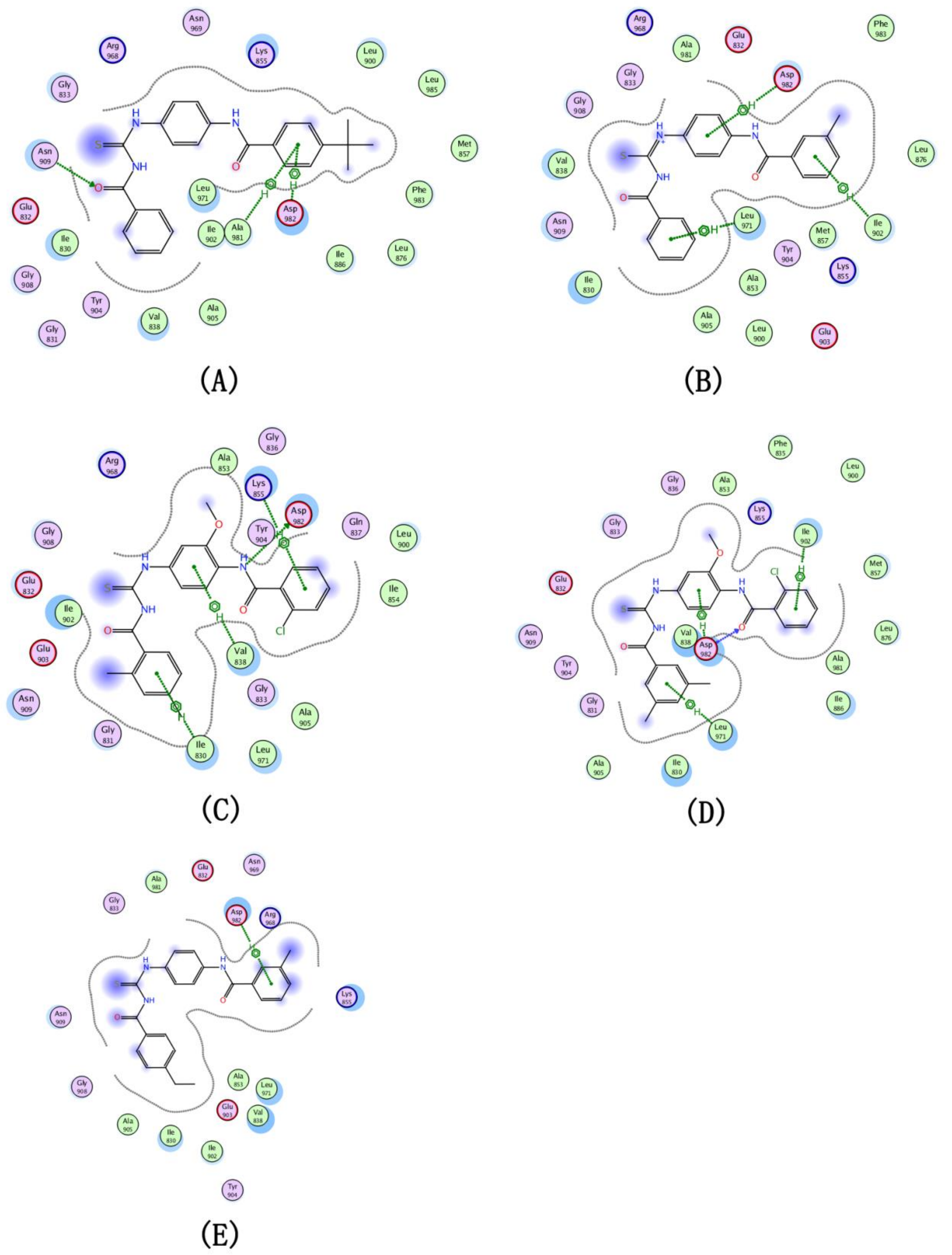

Figure S1. Graphical representations of the binding patterns for compound (A) TP-C3-2, (B) TP-C3-3, (C) TP-C3-21, (D) TP-C3-22 and (E) TP-C3-47. All diagrams were generated based on the average structures from the last stable 3 ns MD simulations.

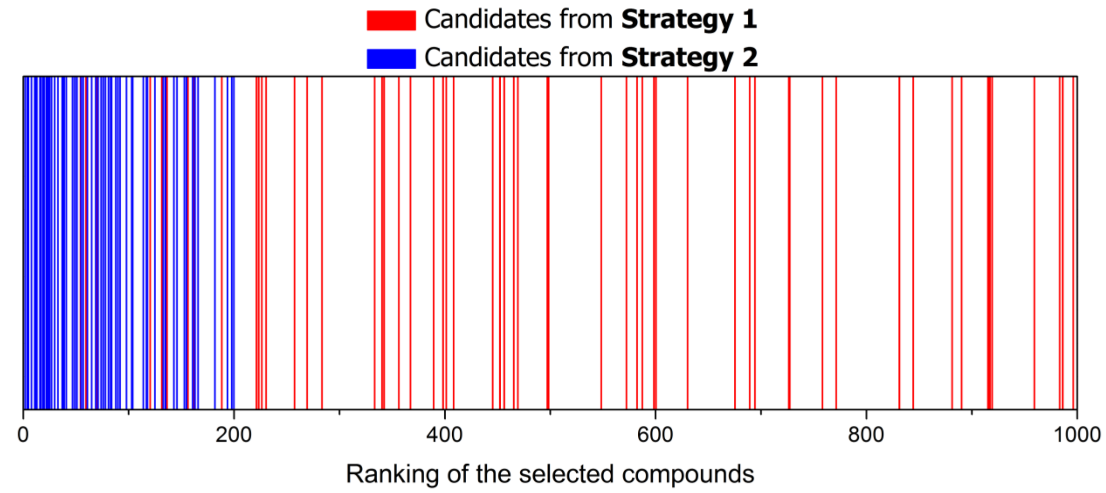

Figure S2. Schematic representation of the rankings of the selected test compounds from both **Strategy 1** and **Strategy 2**.

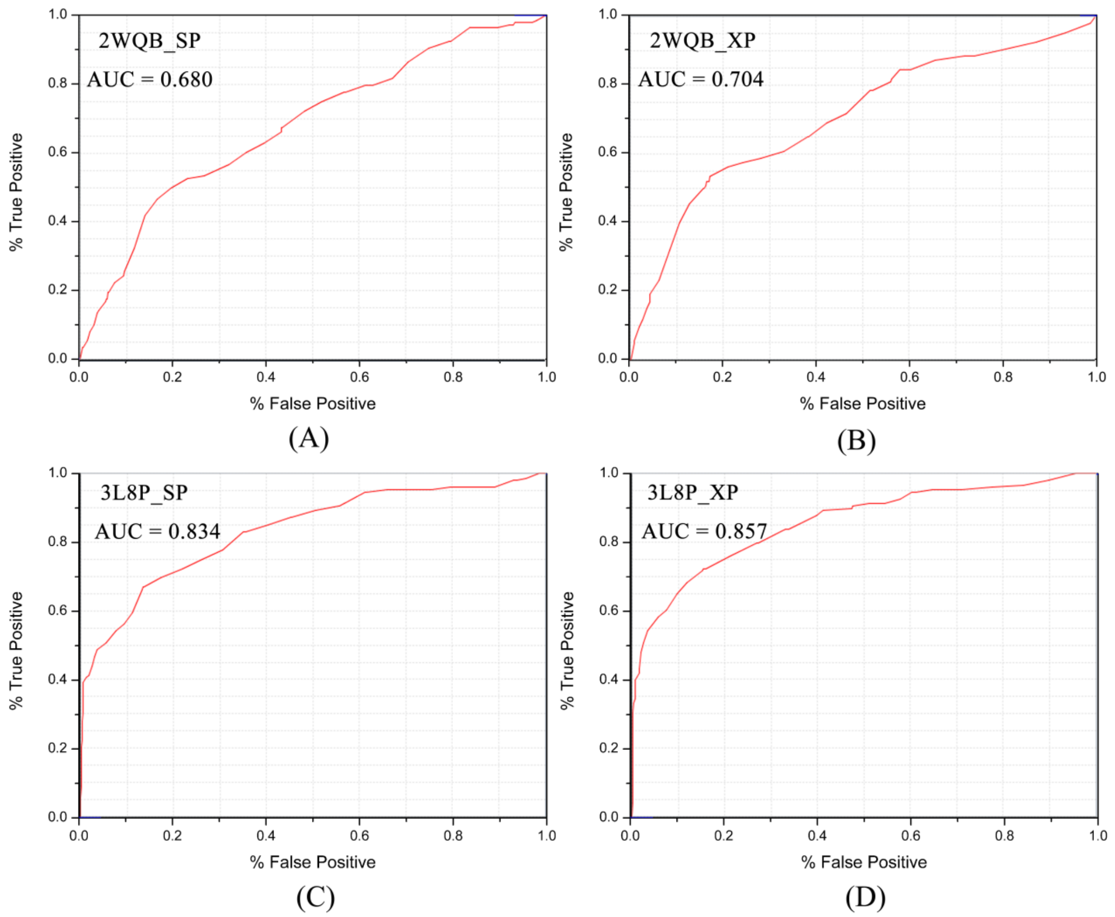

Figure S3. Comparison of the ROC curves based on docking scores for two different crystal structures. (A) PDB entry: 2WQB, Glide SP docking, (B) PDB entry: 2WQB, Glide XP docking, (C) PDB entry: 3L8P, Glide SP docking and (D) PDB entry: 3L8P,

Glide XP docking.

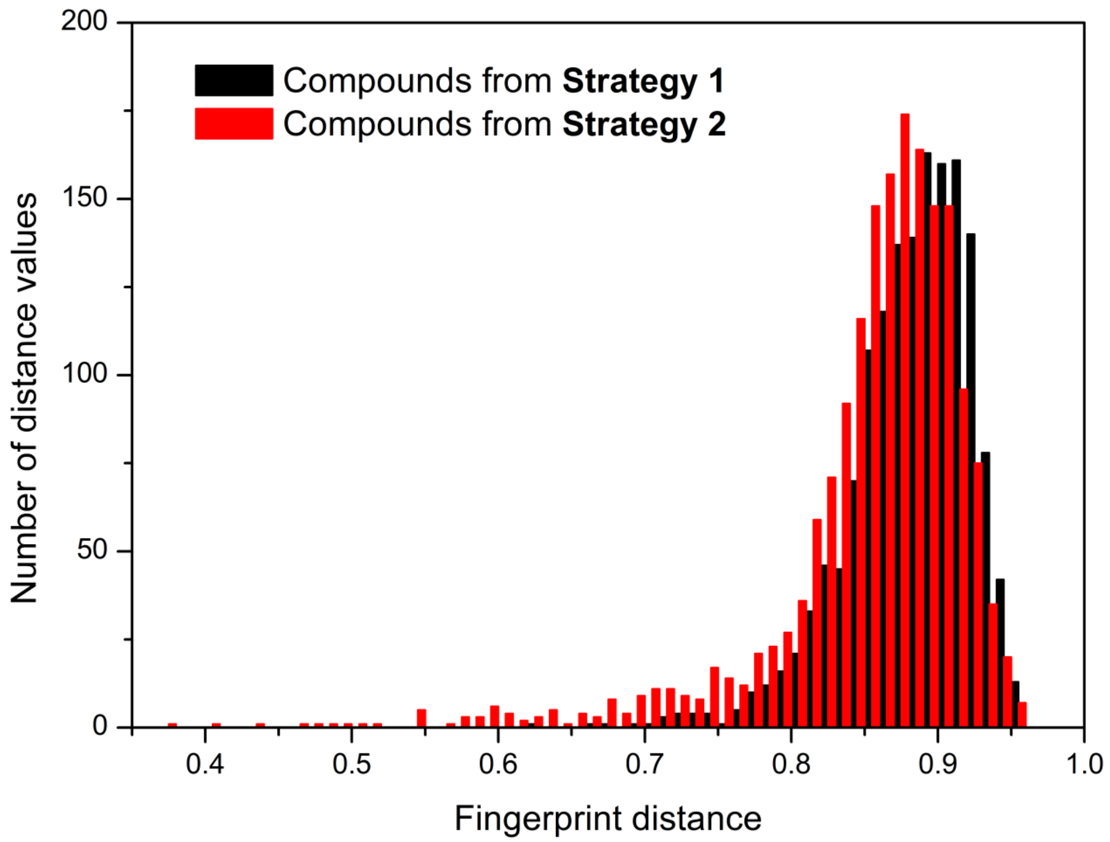

Figure S4. Distributions of the fingerprint distance for the molecules from **Strategy 1** and **Strategy 2**.
